# Clovis point allometry, modularity, and integration: Exploring shape variation due to tool use with landmark-based geometric morphometrics

**DOI:** 10.1371/journal.pone.0289489

**Published:** 2023-08-16

**Authors:** David K. Thulman, Michael J. Shott, Alan M. Slade, Justin P. Williams

**Affiliations:** 1 Department of Anthropology, George Washington University, Washington, DC, United States of America; 2 Department of Anthropology, University of Akron, Akron, Ohio, United States of America; 3 Texas Archaeological Research Laboratory, University of Texas, Austin, Texas, United States of America; 4 Unaffiliated, Rhode Island, United States of America; Sapienza University of Rome: Universita degli Studi di Roma La Sapienza, ITALY

## Abstract

Landmark-based geometric morphometrics (LGM) is most often used in archaeology to characterize and differentiate groups of artifacts, but it can be used for much more. We demonstrate LGM’s power to uncover new insights by exploring stone-tool allometry, modularity, and integration using a sample of 100 western North American Clovis points. Here, allometry concerns how stone tools change in shape as their size changes through their use-lives, and modularity and integration concern how the constituent parts of a tool work together. We show that Clovis points are surprisingly complex tools. When their blades and hafts are defined technologically, rather than arbitrarily, they unambiguously exhibit allometry, and their hafts and blades are modular and highly integrated. We use these analyses to further explore questions about Clovis points, including the differences between cache and non-cache points. Finally, we use heuristic haft-size categories to examine functional constraints on the shape and size of hafts and blades. This work illustrates the importance of using accurate measurements of point components rather than estimates or proxies, which can lead to unfounded inferences. These analytical approaches and accompanying R code are easily transferable to other research questions of stone-tool use.

## Introduction

Stone-tool analysis is often the major, or only, focus of archaeologists on early prehistoric societies, due to the paucity of the archaeological record for reasons of preservation and small populations. In North America, that is the case for the Paleoindian Clovis culture (ca. 13,000–12,700 cal. BP), where most Clovis artifacts are stone tools [1:1, 2]. Using two-dimensional landmark-based geometric morphometrics (LGM), we examine aspects of Clovis points from initial manufacture to ultimate discard or loss. Whether projectile armature or knife or both [2, 3], the Clovis point garners outsized attention and focus. The term “point” is used here because the tool is pointed on the tip and to avoid functional interpretations. We start the inquiry on a significant feature of Clovis points in the archaeological record: Whether the shapes and sizes of points and their component parts (proximal hafts and distal blades) changed from initial design through discard or loss, and if so, how that change can be understood in functional and life-history contexts. In short, we examine whether Clovis points were subject to the phenomena of allometry, modularity, and integration from our dataset of 100 points collected from cache (n = 26) and non-cache (n = 74) sites west of the Mississippi River (Tables 1 and S1 and S1 Fig). Caches are special Clovis site types, such as burials or provisioning loci, that “consist of artifacts or materials in useful condition and forms that appear to have been set aside for later use” [4:1, 5].

**Table 1:** Number of points from sites used in the analysis, including state, site type, and citations.

Site	State	Number	Site Type	Citation
Escapule	Arizona	1	Non-Cache	[6]
Lehner	Arizona	10	Non-Cache	[7]
Leikem	Arizona	1	Non-Cache	[8]
Murray Springs	Arizona	3	Non-Cache	[9]
Naco	Arizona	6	Non-Cache	[10]
Shaldack	Arizona	1	Non-Cache	[11]
Dent	Colorado	2	Non-Cache	[12]
Drake	Colorado	12	Cache	[13, 14]
Fox	Colorado	1	Non-Cache	[15, 16]
Greeley	Colorado	1	Non-Cache	[16]
Kersey Gravel Pit	Colorado	1	Non-Cache	[16]
Rummels-Maske	Iowa	3	Cache	[17, 18]
Simon	Idaho	4	Cache	[19, 20]
Kimmswick	Missouri	2	Non-Cache	[21]
Anzick	Montana	5	Cache	[22–24]
Blackwater Draw	New Mexico	18	Non-Cache	[25–27]
Domebo	Oklahoma	2	Non-Cache	[28]
Jake’s Bluff	Oklahoma	2	Non-Cache	[29, 30]
Dietz	Oregon	1	Non-Cache	[31, 32]
Lange-Ferguson	South Dakota	2	Non-Cache	[33]
Gault	Texas	10	Non-Cache	[34, 35]
Miami	Texas	2	Non-Cache	[36–39]
East Wenatchee	Washington	2	Cache	[40–42]
Colby	Wyoming	3	Non-Cache	[43]
Casper	Wyoming	1	Non-Cache	[44]
Carter-Kerr-McGee	Wyoming	1	Non-Cache	[45]
Hell Gap	Wyoming	2	Non-Cache	[46]
Sheaman	Wyoming	1	Non-Cache	[47]

**Fig 1:**
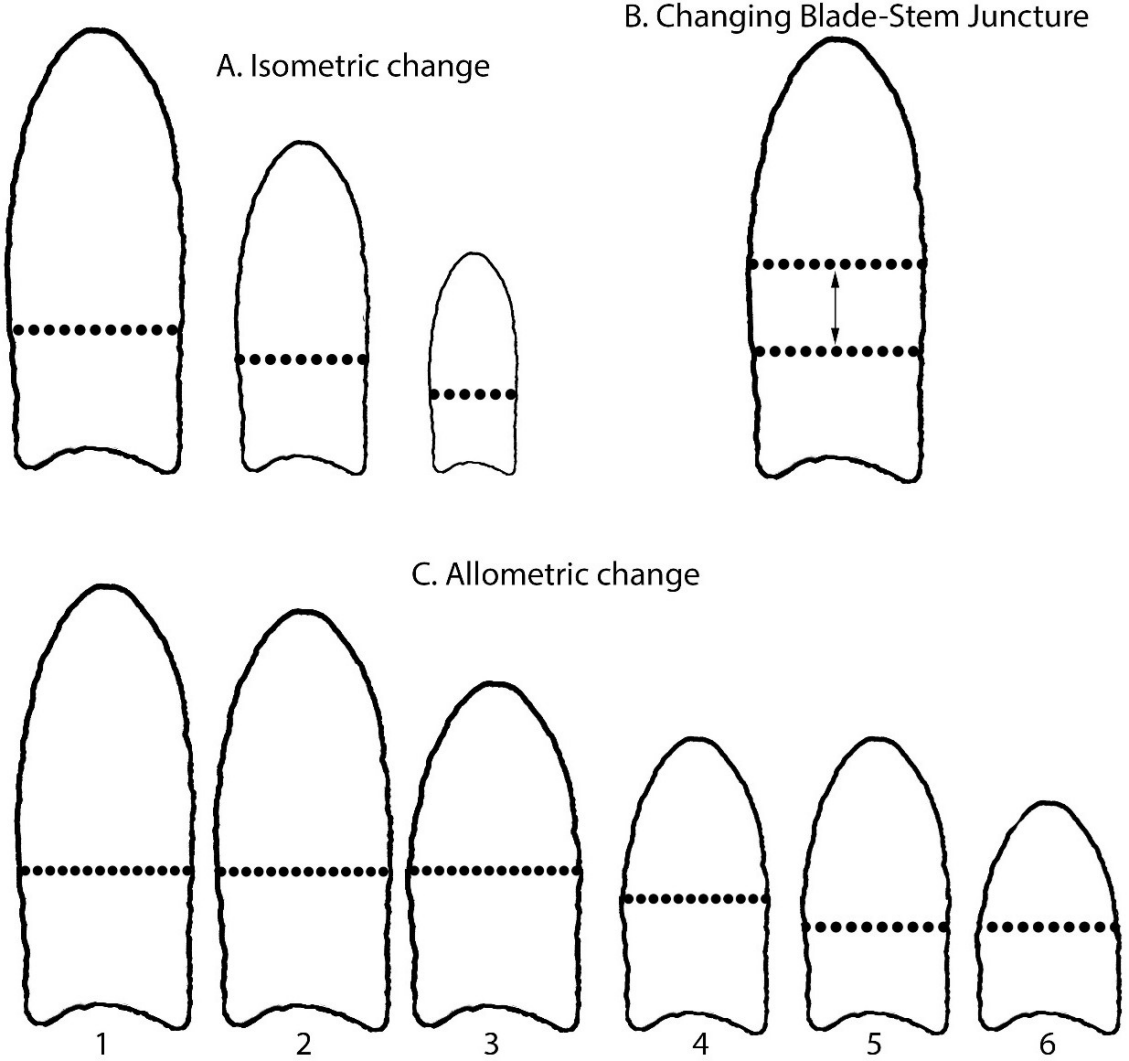
Possible Clovis point shape changes. (A) Isometric change. (B) Moving the haft-blade juncture alters blade-length without affecting overall length. (C) Possible sequence of resharpening, reworking, and haft-blade juncture changes during use-life.

Allometry is discussed in more detail below, but succinctly: if an artifact shape changes non-uniformly as its size changes, such as resharpening just the blade of a point but leaving its haft unchanged, the change is allometric (Fig 1C). If a tool’s shape changes uniformly with changes in its size, perhaps by resharpening and reworking the tool around its circumference, the two-dimensional shape-change is isometric (Fig 1A) [48]. Allometric effects can manifest as a change in the entire shape or the relative proportions of the tool-components’ shapes with change in overall size of the tool. Next, we explore related concepts of modularity and integration of Clovis point blades and hafts, which provide insight into how Clovis people thought about point-design and rejuvenating a dull or broken tool. Modularity refers to the different functional parts of a tool. Although created from a single stone, hafts and blades are modular, in that they are designed for different functions, but also integrated so they function as a single tool. Modularity analysis can reveal how stone tools were designed to be used safely and effectively from manufacture through discard or loss as the blade was resharpened or repaired. Integration is how the functional parts work together. Integration analysis explores whether the haft and blade covary in concert or independently. We look at each of these properties statistically to determine whether they are present in Clovis points, and then consider the concepts together to better understand how the points were designed and used. These concepts also are used to interpret statistical differences between cache and non-cache points. Lastly, we use the allometry, modularity, and integration results along with linear dimensions and centroid sizes to understand the design constraints on hafts and blades, i.e., how big or small a haft must be to safely and effectively hold different-sized blades.

In North America, chipped stone points are typically composite tools made of two modules: a proximal haft, which is attached to an organic haft, such as a handle or shaft (we use the term “handle” to distinguish the organic haft from the point haft), and a distal working end, which may be used for cutting, scraping, piercing, or other function (we use the term “blade” as a catchall designation [49:179–180]. We infer blades were sharpened or repaired, whereas hafts may have been reworked to repair damage, maintain edge continuity, or fit new handles. To clarify the discussions that follow, we define *resharpening* as the removal of flakes from dull or damaged blade edges to create new sharp edges. In contrast, *reworking* is the removal of flakes to change the haft’s shape or size without sharpening, although distal edges of a haft may be sharpened and incorporated into the blade.

The distinction between blade and haft modules is usually obvious. For example, in Early Archaic (ca. 11,500–8,000 years BP) notched or hafted points in the southeastern US, notch size and haft width appear set within a narrow range because the blade-haft intersections cannot be modified without fundamentally altering the haft shapes in obvious ways [50]. This is not the case with Clovis and other lanceolate-shaped points in which the transitions from haft to blade are often not obvious. We agree with the general consensus that the distal extent of proximal lateral grinding or abrasion marks the transition and divide hafts and blades accordingly (dotted lines in Fig 1) [35, 51, 52:38,cf. 53]. Despite the difficulties with identifying the transitions, we think the lengths of lateral grinding must be measured rather than estimated. Only by measurement of each point can one confidently divide the haft from the blade that matches the point’s technology and be confident in the analyses and inferences. Using nontechnological criteria to define blades and hafts, such as using constant percentages of overall point size [e.g., 54] or a single length to define the haft [e.g., 55] masks the variation in point sizes and shapes produced in the past, leading to potentially skewing analyses and inferences.

## Incidence of allometry, modularity, and integration in archaeology

The phenomenon of allometry is generally well-accepted in the analysis of chipped stone points and other tool types [56:82–83 and references cited therein] and probably accepted intuitively by most analysts [57, 58], but modularity and integration are rarely discussed. Allometry is manifested as the tool is reduced in size during repair, resharpening, or refurbishing to satisfy a different function. Allometric variation is documented in flake tools, [e.g., 59–62] and especially in bifaces [55, 63–70: 93–99, 71–74:108–120, 75]. That bifaces should be particularly susceptible to allometric reduction is no surprise; on the contrary, their design, use and the damage they often suffer suggest that point blades, especially tips, require much more repair by resharpening than do other segments or modules [65:163]. In one experimental study, for instance, all tested points suffered damage on blades but few on hafts as well [76:204]. More extensive damage in other experiments also was concentrated on blades [56:Fig 1]. Any similar prehistoric damage, whether repaired in hafts or not, would require resharpening and therefore reduction concentrated on point blades. The result would be disproportionate size reduction on blades compared to other modules like hafts.

As above, most lithic analysts, including many Paleoindian ones, interpret much of the variation expressed in Paleoindian points as allometric. They agree, that is, that “Abundant experimental and allometric studies on projectile points have shown that…major size and shape changes most frequently occur in the point blade, especially in its length” [77:163], a conclusion also reached in Buchanan’s [64] classic Folsom study.

In several studies over the last decade, Buchanan and colleagues have argued that Clovis points do not show significant effects of allometry, concluding: Clovis points were not often resharpened, which was only a minor source of Clovis point variation [78:19]; Clovis points were resharpened allometrically but blades were likely resharpened isometrically despite the authors’ own general conclusion that in their dataset Clovis “allometric relationships were produced primarily through resharpening” [79:11]; resharpening did not result in more shape variation in blades than bases [54:728, see also 80:5], and resharpening and allometry may occur but that fluted-point types preserve their original shape against allometry’s minor effects [81:356]. To resolve the difference between the general consensus of allometry’s common expression and Buchanan and colleagues’ positions that allometry either does not occur or has minimal effects, we undertake the first explicit test of allometric variation in Clovis points using LGM methods and computer programs and statistical methods designed specifically to test for allometry in shape-data.

### What is allometry and how is it measured?

The concept of allometry is well-developed in biology, where it is used to describe an organism’s growth or evolution through time, but it is not exclusively a biological concept. It is important to note that whereas allometry is simply a scaling relationship of a changing variable (e.g., shape, size, or number), the explanations for the phenomenon are discipline-specific. The classic example of allometry in biology is the way size and shape of the human head changes from birth to maturity relative to other body parts [82]. In contrast, isometry occurs when size and shape are independent, meaning shape change is not correlated with size change [48]. Changes in allometric rate and timing can make profound differences in an organism’s final phenotype [83]. In contrast to biological growth, allometric changes in chipped stone tools are in the opposite direction, tools becoming smaller through time, but mathematically the processes are identical [65, 84, 85]. LGM data are well-suited for allometry analysis because it treats shape independently of size. Shapes are rescaled to minimize size differences, so the shapes of a large Clovis cache point can be compared with a small non-cache point without the confounding effect of size. Allometry in LGM is identified by a line of best-fit in multivariate regression of shape on log-transformed size variables, usually centroid size (CS) [48, 86], but allometry also can be studied in traditional tool dimensions, such as weight and length [e.g., 85].

In archaeology, allometry is surprisingly complex, with conceptual difficulties related to manufacture, use, and discard behaviors. In biology, researchers know how extant organisms grow and can fit their shapes in a growth continuum, making allometric inferences straightforward [87]. In contrast, we rarely know the starting shape or size of a prehistoric point for certain [e.g., 88, 89], nor do we know whether the point we excavate was discarded at exhaustion or lost beforehand. This situation is more akin to paleontologists trying to reconstruct growth histories of odd extinct organisms with sparse or noncontinuous data [90]. Some of the difficulties in interpretation are explained below.

Allometric concepts have been used to infer patterns of tool use, seasonal movements, and distances to quarries [63, 84, 91]. But allometry is also an important consideration in the analyses of prehistoric social patterns [92, 93]. Point shapes are commonly used to infer prehistoric social relationships and organization or migration, under the general assumption that related social groups share social behaviors, such as making similar point shapes [94–97]. Changes to the initial shape of a tool through damage, resharpening, or reworking potentially skew the statistical analyses from which social relationships are inferred [98, 99]. Entire point shapes are the most commonly used proxy for social relatedness, so ensuring shape variation created during initial manufacture and subsequently changed through resharpening are not conflated is important [93]. If shape change from reworking or resharpening is isometric, then shape analyses without accounting for allometry will be unproblematic. But if allometric shape change is significant, then conflating equifinal causes of shape variation is likely.

Allometry in points can be analyzed in several ways. The simplest is to examine whether the entire point shape varies with the entire point size. But allometry can also be analyzed using components of the entire point-shape. In this case, the hafts and blades of the entire point are natural components to consider because they are intentionally created parts of the point’s design. Examining the effects of the blade and haft on the point’s allometry can provide insight into initial design and subsequent use of the tool.

#### What are modularity and integration?

The concepts of modularity and integration are closely related but not simply two ends of a continuum [100]. We naturally think of the blade and haft modules of Clovis and other prehistoric points as integrated into a functional whole [101–103:2]. This common conceptualization of modularity and integration concerns function–how the two modules contribute to the functionality of the tool. A knife with a handle and a blade is a good analogy for these concepts. The handle and blade are independent modules of the knife, and each module can be analyzed independently. But, although independent, the modules are integrated and function together for the knife to work properly. One would never use a handle without a blade and using a blade without a handle could be dangerous or ineffective. Also, different blade shapes can require different handle types. In contrast, a pencil with an eraser is modular but not integrated. Each module can function independently with no loss of effectiveness. We may seek to understand whether a different tool design would be more or less effective, or how module designs evolved through time or varied across space, and from these infer why the human choices were made. But in the end these inquiries concern tool function. Modularity and integration are used differently in shape analyses, where the goal is to understand how and the degree to which module shapes vary independently. In biology the concepts are used to investigate developmental, genetic, or functional linkages within and among taxa (see [104] for examples). In archaeology, the concepts can provide insight into tool function and use-life.

Here, we are interested in the relative independence of the Clovis blade and haft components, i.e., the relative degree to which their shapes covary [105:591] because that provides insight into how the tools were used and changed through their use-lives. Modularity means components have greater within-component than between-component covariation. But modularity is not an either-or condition; shapes can be more or less modular [100:121]. A shape structure is integrated if its components are correlated. Perfect integration means the variation in a small region of the shape would perfectly predict the rest of the shape [106:44]. To illustrate the concept for Clovis points, perfect integration hypotheically could mean every time the haft changes by 1% (however that might be measured), the blade changes by 5%. The changes are not necessarily isometric because change may not be uniform, but they are perfectly integrated. Shape components can be modular and not integrated, integrated and not modular, neither integrated nor modular, and both *modular and integrated*.

This last possibility may seem counterintuitive but can occur in part because of ways modularity and integration are tested; the modularity signal will be relatively stronger or weaker depending on the strength of integration [100:597, 105]. Allometry can contribute substantially to integration of shape components [105:594]. Even if modularity is significant, strong signals of allometry affecting most or all of the structure can produce strong integration [106:47] and obscure strong but relatively weaker modular structure [107:416]. A structure with strong within-module covariation but even stronger between-module covariation may result in an erroneous inference of no modularity.

## Material and methods

Our dataset (Tables 1 and S1) came from 2D images collected by Slade [108] and Williams [109, 110] by scanning or photographing original artifacts or casts, obtaining images from museum staff or archaeologists, or from the internet. We chose to use 2D mainly to make our analyses more directly comparable with earlier work but also because the 2D data was readily available. We suspect the third dimension–thickness–would not have a significant effect on the results, but this is a hypothesis worth testing in future work. Other than abrasion length, which was always taken from actual points or high-quality casts, linear dimensions were usually measured by calipers on points or casts, but sometimes from images. Using high-quality casts does not affect the accuracy of the linear measurements [64] or the detection of lateral abrasion. Casts analyzed by Shott and Otárola-Castillo [56] retained clear evidence of edge abrasion that correlated with independent measurement of the haft-blade juncture on the corresponding stone points by Hunzicker [111]. Images deemed too low quality for accurate landmark placement or linear measurement were not used. Linear measurements taken from well-scaled and scanned images have been found to produce accurate results [109, 112]. The lateral extent of abrasion was identified by lightly running fingers along the edges several times to find where the abrasion ended and then measured with calipers. Points that did not have unambiguous transitions were not used. All lateral abrasion measurements were done by Slade to eliminate inter-observer variation. We defined the haft-blade juncture as the longest abrasion-length when it differed on each side. Data on individual points, including site name and type, haft-length, haft-width, blade-length, maximum-length, heuristic haft-size class, and the ratio of haft-length to maximum-length are in S1 Table.

**Fig 2:**
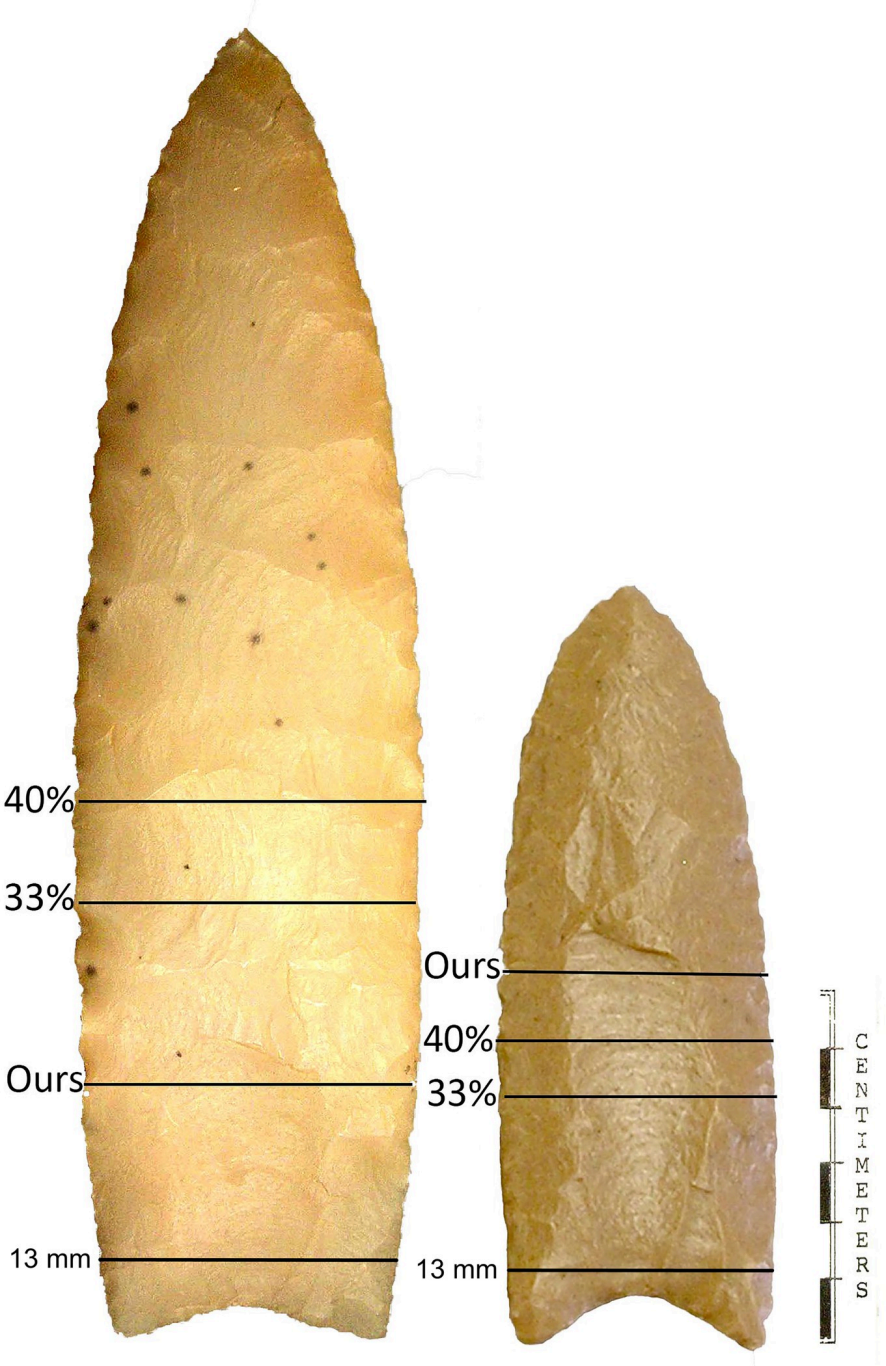
Comparison of haft/blade junctures. Points from the East Wenatchee, Washington (left, 21%) and Leikem, Arizona (right, 48%) sites showing differences in our measured and Buchanan and colleagues’ estimated haft-blade junctures at 33% and 40% of total length measured from tip to the basal ears and Smith et al.’s 13 mm measurement. Images from Slade [114].

Fluted points, including Clovis points, have been analyzed with LGM frequently, although techniques vary [55, 56, 81, 93, 94, 96, 98, 99, 113]. Here we focus on two approaches, ours and those used by Buchanan and colleagues in several publications. Most of the details are the same, but we mainly differ in how the hafts and blades are defined, which makes a substantial difference in analysis, results, and inferences. The question is which approach is a better proxy for capturing the haft and blade shapes: our actual measurements based on technological indicators of blade/haft transition, or Buchanan and colleagues’ approximations based on percentage of total point length to define the blade/haft dimensions on all points. We find the approach taken by Smith et al. [55:103245] to use a single standard measurement (13 mm) of the proximal end of each point to define the “bases and blade portions” of fluted points to be useful in some analyses, but neither are appropriate proxies for hafts and blades (Fig 2). The analytical effects of the different LM placement strategies are discussed in more detail in the Discussion.

**Fig 3:**
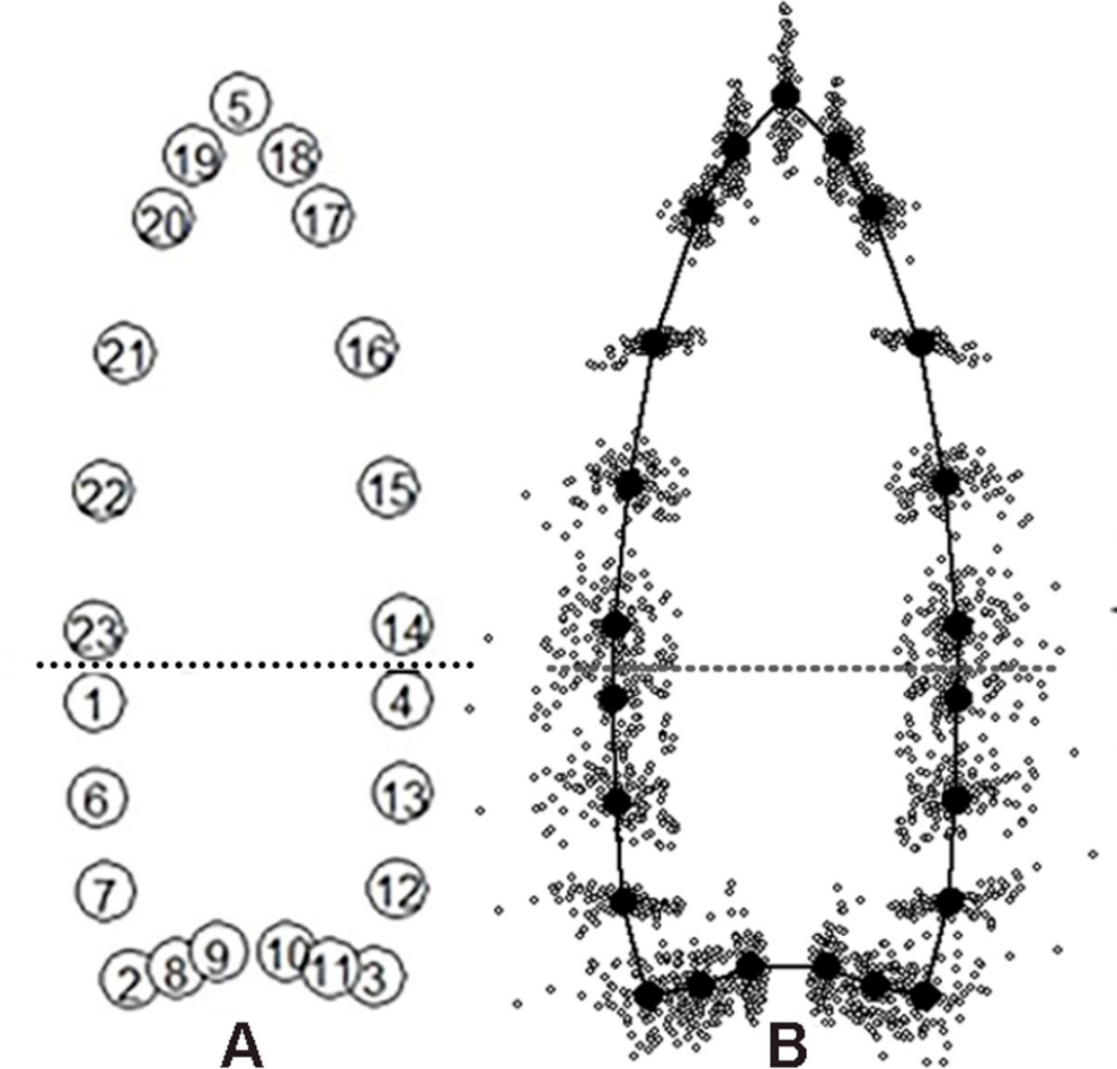
LM configuration and LM distributions after GPA. The dotted horizontal lines show the stem and blade juncture.

We defined the outline of the points with 23 total LMs using tpsDig2 v. 2.31 [115]: five regular LMs at the blade-haft juncture (LMs 1, 4), haft ears (LMs 2–3) and blade tip (LM 5) and five connecting curves with a total of 18 evenly spaced semi-landmarks between them, which were converted to regular LMs in tpsUtil v. 1.76 [115]. Three of us (DT, MS, JW) placed LMs and semi-landmarks, estimating LM locations for small breaks on tips, ears, and edges, and the results were averaged. Points with large breaks were not used. The 23-LM configuration (Fig 3A) mimics the 23-LM configuration used by Buchanan et al. [54:Fig 2]. We did not slide semi-landmarks because we do not see an anthropological reason justifying the practice. In contrast, Buchanan and colleagues [54:Fig 2] defined the outline of points using 3 LMs at the tip and 2 ears. Then semi-landmarks were presumably evenly placed along the outline with tpsDig2 and converted to LMs. We note that the LM configuration in the 2018 publication is different from those used earlier [78, 94, 116], where the program MakeFan6 [117] was used. MakeFan6 creates equally spaced lines perpendicular to a base line that intersect the edges for LM placement. Both techniques are perfectly acceptable but produce slightly different LM configurations that are not precisely comparable.

LGM analyses employ entire shapes as single multivariate units of spatial relations and interrelations of the landmarks that define them [48]. LGM eliminates the need to rely on a set of “traits”, such as linear dimensions, angles, ratios, or categorical data, which usually are limited shape descriptors [56, 106, 118–120]. Our 100 Clovis point shapes were translated, rotated, and rescaled to unit size for comparative analyses through generalized Procrustes analysis (GPA), which minimizes the distances between homologous LMs on each shape through a generalized least squares process [120]. After GPA, the shapes are orthogonal to (i.e., independent of) size unless allometry is present. Thus, LGM allometric analysis determines whether size covaries with shape after GPA, i.e., whether the shapes of the Clovis points, or their parts, change nonuniformly as points get smaller through resharpening or reworking.

## Results

### Allometry analyses

In LGM analyses, allometry multivariate regression tests for a relationship between the dependent differences in shape on independent differences in size [105]. CS is the standard size proxy in LGM analyses [106:47], although other measurements may also be appropriate [85]. We use centroid sizes of the entire point (CSE), blade (CSB), and haft (CSH) shapes, which are calculated as the square root of the sum of the squared distances from all the LMs defining the shape to the shape’s center [48, 83]. The natural logarithm of CS (lnCS) was used in all analyses. Significance of the covariation of size and shape is tested with a nonparametric permutation test based on Goodall’s [121] *F* statistic.

Procrustes-aligned entire point, blade, and haft shapes were regressed upon their respective lnCS using the generalized linear model (procD.lm function) in the *geomorph* package [122] in R 4.2.2 [123] run in RStudio v.2022.12.0+353 [124]. The presence of allometry was tested by comparing the regression slopes against a null model of isometric change in a Procrustes ANOVA performed using the sum of squared Procrustes distances [121]. Isometric change would have a slope of zero, i.e., no change in shape as size changed, whereas allometric change would have a non-zero slope. *Geomorph* implements a residual randomization permutation procedure (*RRPP*) that randomizes model residuals to estimate a probability distribution for the interaction effects [125–127]. Among its many benefits, *RRPP* facilitates nonparametric ANOVA analyses. R code used in the analyses and to create most of the figures is in S1 File.

The first allometry test is straightforward: whether the entire point shape shows an allometric signature. It does, as do the haft and blade modules. The results are highly significant for the entire shape (*F* = 55.22; *df* = 1, 98; *p* < .001), the haft shape (*F* = 26.95; *df* = 1, 98; *p* < .001), and the blade shape (*F* = 69.67; *df* = 1, 98; *p* < .001), and we confidently reject the null hypothesis of “no allometry” (full results in S2 Table 1 in S2 Table). The ANOVA results also show most of the shape changes occur in the blades. The r^2^ coefficient of determination is a measure of the amount of shape variation explained by changes in size. Here, 36% of shape variation in the entire point is explained by overall size, and 22% of the haft-shape change is explained by haft-size. In contrast, 42% of blade-shape change is explained by changes in blade-size, supporting an inference that most allometric change in Clovis points is due to blade resharpening.

### Allometry of cache and non-cache points

**Fig 4:**
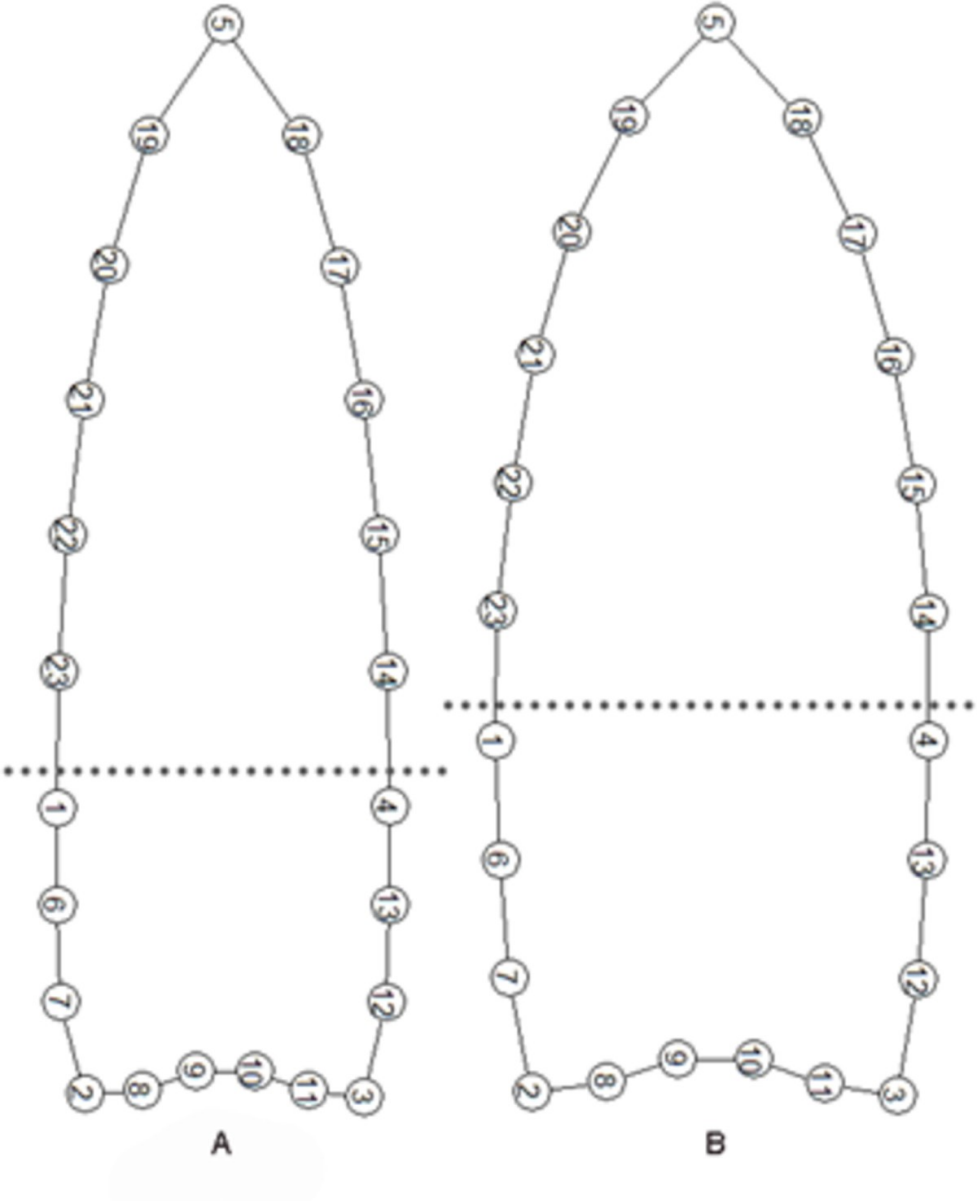
Mean shapes for (A) cache and (B) non-cache points. Haft-blade junctures indicated with dotted lines.

**Fig 5:**
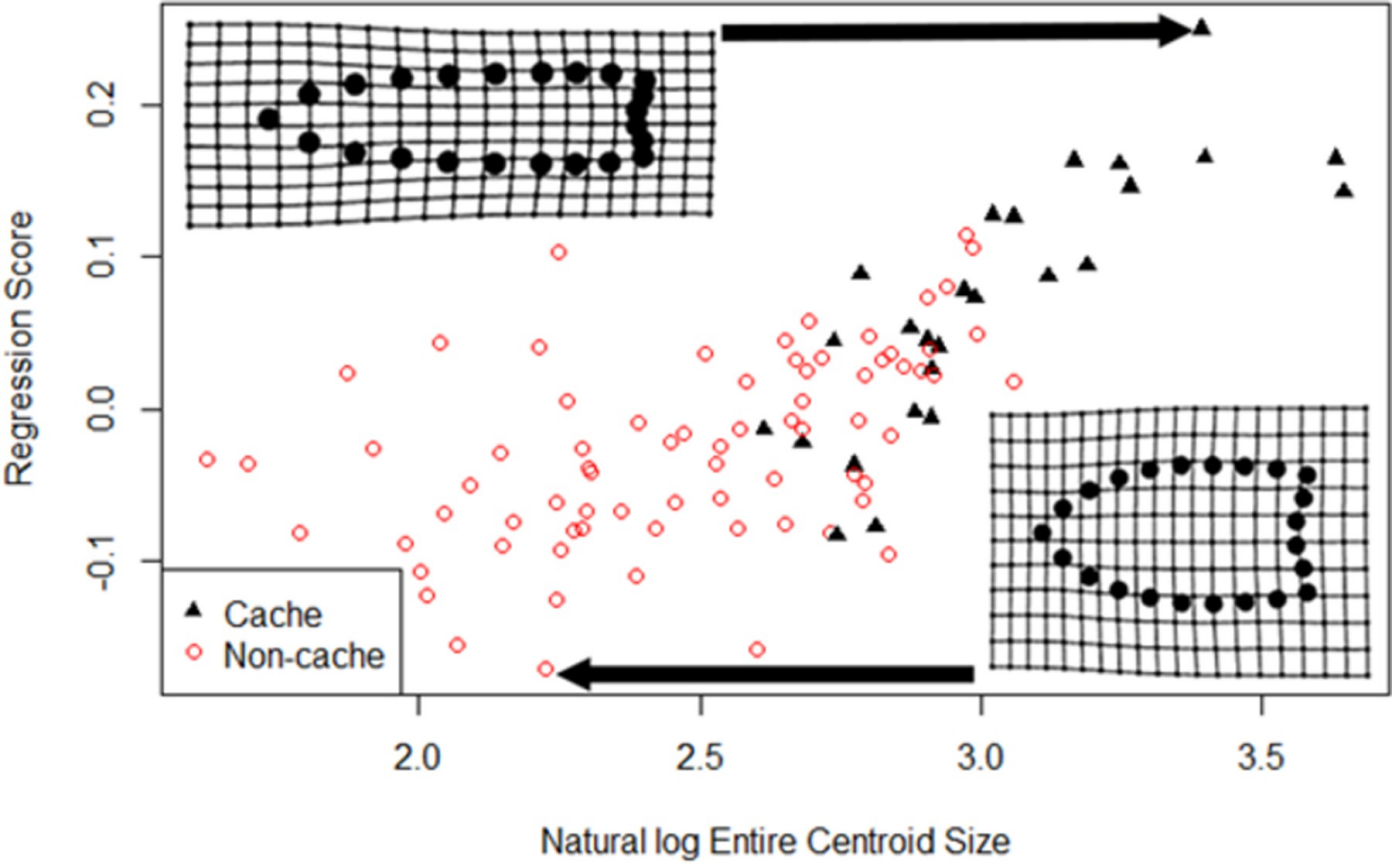
Plot comparing shape changes (regression scores) v. lnCS of cache (black triangles) and non-cache (red open circles) points. Estimated shapes in grids (function picknplot) associated with the points with highest and lowest regression scores are shown.

Exploring further, we examined differences between cache and non-cache points. Caches are classic Clovis site types (e.g., [4]) that others have analyzed to determine whether either or both Clovis point shapes or allometries are different. We relied on previously published cache site identifications (S2 Table 1 in S2 Table). Buchanan, Kilby, et al. [79:4] inferred the shapes of cache and non-cache Clovis points were not significantly different. We found the opposite; entire, blade, and haft-shapes were all highly significantly different (Entire: *F* = 16.87; *df* = 1,98; *p* = .001; Blade: *F* = 22.78; *df* = 1,98; *p* = .001; Haft: *F* = 12.97; *df* = 1,98; *p* = .001; S2 Table 2 in S2 Table). Fig 4 shows mean cache and non-cache shapes and illustrates the differences in the positions of their haft-blade junctures. They also inferred [79:4] the allometries of cache and non-cache Clovis points were not significantly different, meaning they were resharpened and reworked in the same ways. We tested the differences in allometries by comparing the common allometry of all 100 points with the unique allometries of the cache and non-cache points. We found that allometry patterns of cache and non-cache entire point shapes were significantly different (*F* = 9.843; *Resdf* = 97,96; *p* = .002; S2 Table 3 in S2 Table). Fig 5 plots the unique allometries of cache (black triangles) and non-cache points (red open circles) for entire shapes on their lnCSs (function plotAllometry). Shapes are described by Regression Score, which “is the shape variable that has the maximal covariation with. .. log-transformed centroid size” [48:122]. In *geomorph*, regression scores are projections “of data on [a] normalized vector that expresses the covariation between shape and the regression coefficients for size.” [48:122, 123]. Plots of regression scores versus lnCSs capture both the projected variation due to size and the residual variation, which is typically the error component. Here, some error can be understood as variation due to other factors, which could include region, raw material type and package-size, site function, time period, tool-function differences, or statistical error. These findings complement and do not contradict our earlier finding that Clovis points overall show robust allometries in their entire shapes, blades, and hafts (S2 Table 1 in S2 Table).

### Modularity and integration analyses

Modularity and integration in *geomorph* are evaluated to identify “exceptional” correlations relative to some H_0_. Modularity is assessed with a covariance ratio (CR), measured against a null of no modularity using a permutation test. The CR statistic is the ratio of between-modules and within-module covariances. Thus, a CR statistic of 1 means the covariance between and within modules is the same. A CR statistic below 1 means the covariance within modules is higher than between modules, which translates to a signal of modularity [104]. Our CR is 1.0018, which >1, but the distribution is skewed and the *p* = .017 indicates the null hypothesis of “no modularity” should be rejected).

For integration we use a Multivariate Association measure (r-PLS), and the best of those is Partial Least Squares (*geomorph’s* integration.test function). We ask whether there is greater covariation between modules than expected by random association of modules. Unlike the modularity test, integration.test does not concern within-module covariation and is evaluated by shuffling the rows (objects) of one module while leaving the other module the same [104]. The null hypothesis is no integration. Our data had a r-PLS value of 0.943 and *p* = .001, with an effect size of 7.5694, which indicates the blade and haft are very strongly integrated (Dean Adams, personal communication 2020).

Our interpretation is that Clovis points have a strong signal of modularity but also very strong integration; these interpretations do not conflict. The allometry and modularity results indicate much of the variation in Clovis points lies in changes in location of the haft-blade junction. Strong integration manifests in the fact that Clovis points are readily identifiable by their general lanceolate shape with no dramatic transition from haft to blade regardless of size. Changes in small parts of the point shape are somewhat predictive of overall point shape change. Contrast this with a Paleoindian Dalton point, in which the size and shape of the haft is not predictive of blade size and shape [58, 128, 129].

## Discussion

In sum, our results show that Clovis point shapes produce a strong signal of allometry, and we reject the null hypothesis of isometry (Fig 1A). We conclude that Clovis people made, used, and resharpened the point blades at rates and in manners different from point hafts, and the distribution of Clovis point shapes we find in the archaeological record resulted from intentional discard and unintentional loss of points at varying times in this initial design and resharpening/reworking process. Most of the shape changes are in the blade, although the haft also changes, indicating at least some resharpening was likely. The modular blade and haft shapes are strongly integrated, meaning they were designed, resharpened, and reworked in concert, although not in the same ways. By closely examining how the haft and blade dimensions covary, we can get a better understanding of how these integrated modules were constrained in size and shape during their initial designs and use-lives.

Why do our results differ so markedly from Buchanan and colleagues’? The main reason seems to be the definition of the blade/haft transition. Both analyses divided the points into two modules: a blade and a haft, indicated by dotted lines in Fig 3 and Buchanan et al. [54:Fig 2]. Although the images look similar, especially at the important blade-haft juncture, the criteria for setting that juncture are different. We used specific LMs to define the lateral extents of that transition whereas Buchanan and colleagues did not. Buchanan et al. [54] used a single approximation of the juncture, and we measured it. Measuring along the midline from the point tip (LM 1) to the top of the basal concavity, the Buchanan and colleagues’ haft-length is about 40% of the entire length. This technique ensures the haft and blade will maintain their ∼40/60% relative dimensions regardless of the actual location of the juncture as measured by abrasion length. If a blade was shortened through resharpening but the haft untouched, Buchanan and colleagues’ relative position of the juncture would ensure that the blade was always ∼40% of the entire length, whether that actually happened in the past.

It is also important to note that the LGM estimate of blade-haft juncture in Buchanan and colleagues’ [54] analysis is different from their prior estimates of juncture location that used univariate estimates of haft and blade size. Buchanan and colleagues used an arbitrary estimate of haft-length of 1/3 of the overall length [e.g., 80:5, Table 2]. Because the edge boundaries are curved, 1/3 of their lengths will be near but not precisely the same as 1/3 of the total midline length. Our measured lengths produced a range of values for haft length as a percentage of maximum length (S1 Table). For all data, the mean and median are 32% but ranges from 12–50%. Interestingly, the mean and median of non-cache points is 33%, but cache points are 27% and 25%, respectively. Further, cache points range from 12–46% and non-cache points from 16–50%. Fig 3 compares the measured juncture locations on the cache East Wenatchee (21%) and non-cache Leikem (48%) points (S1 Table) with Buchanan and colleagues’ 33% and 40% estimates, and Smith et al. [55] 13 mm measurement.

**Table 2:** Size ranges of the Clovis points by the heuristic stem-width size categories.

	Number	Stem-width	Stem-length	Blade-length	Maximum Length
**Non-Cache Very Small**	5	16–21	5–8	22–31	27–38
**Non-cache Small**	18	17–24	12–27	10–50	23–73
**Non-cache Medium**	23	25–29	12–40	25–67	39–105
**Non-cache Large**	28	30–40	19–39	37–90	75–116
**Cache Medium**	2	29	27–34	51–52	79–85
**Cache Large**	20	32–40	18–41	51–164	85–186
**Cache-Very Large**	4	41–64	26–48	91–188	117–232

Medium, large, and very large cache point size ranges are included for comparison. Measurements in mm.

It is unclear whether Clovis points were made in a single large size and resharpened and reworked before discard (Pattern 1), several standard sizes and only resharpened before discard (Pattern 2), or many sizes and rarely resharpened before discard (Pattern 3). These three design-and-use patterns are illustrated in Fig 1C. Pattern 1 Clovis points would have been made in a large size (with variation due to raw material, haft size, conflated time-trends, and other local conditions) and got gradually smaller as the working edges of the blades were resharpened and the haft was shortened and then narrowed to maintain the integration pattern. In Fig 1C1, a large point was manufactured, then the blade was progressively shortened through resharpening (1C2-1C3). The point was then removed from the haft, the blade resharpened, haft reworked, and haft-blade juncture moved proximally by sharpening the abraded distal end of the haft (1C4). In 1C5, the point was removed from the haft to move the juncture proximally by resharpening the abraded distal edges of the haft to create a longer blade, and then reattached to the same haft. In 1C6, the blade was resharpened. Then the point was not further resharpened and discarded. The actual sequence for any particular point is unknown and likely variable depending on circumstances. Pattern 2 might involve two original sizes, represented by 1C1 and 1C4. The large size 1C1 would be reduced through 1C3, when it was discarded. The smaller size 1C4 was reduced through 1C5, when its juncture was moved, and the point reused and resharpened until reaching 1C6 when it was discarded. In Pattern 3, 1C1, 1C3, 1C4, and 1C5 represent the initial sizes, which were never reworked and either never or minimally resharpened before discard.

**Fig 6:**
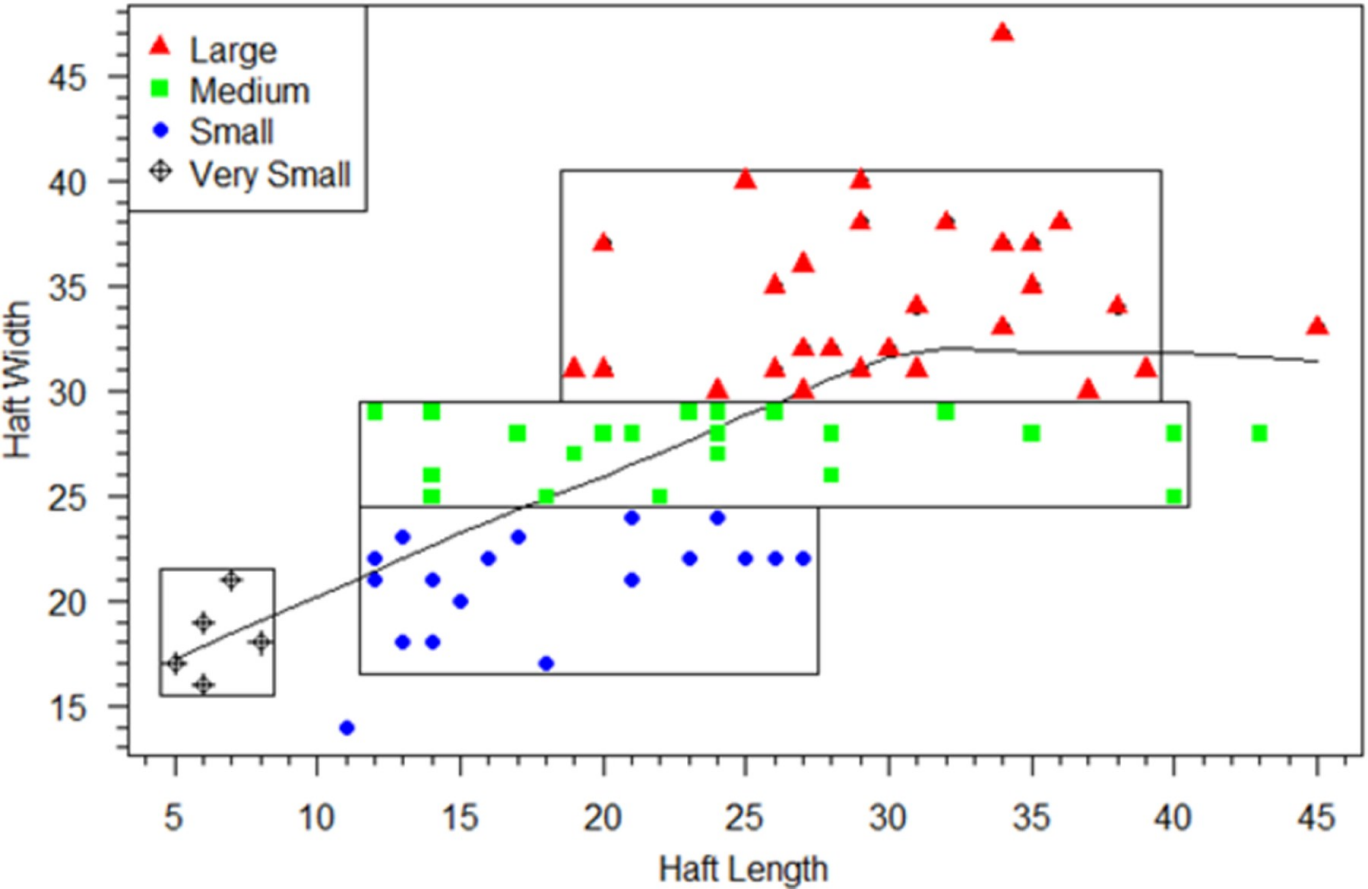
Scatterplot of haft-width to haft-length. LOWESS line and size classes indicated. Boxes enclose the concentrations of points in each haft-width size-category.

**Fig 7:**
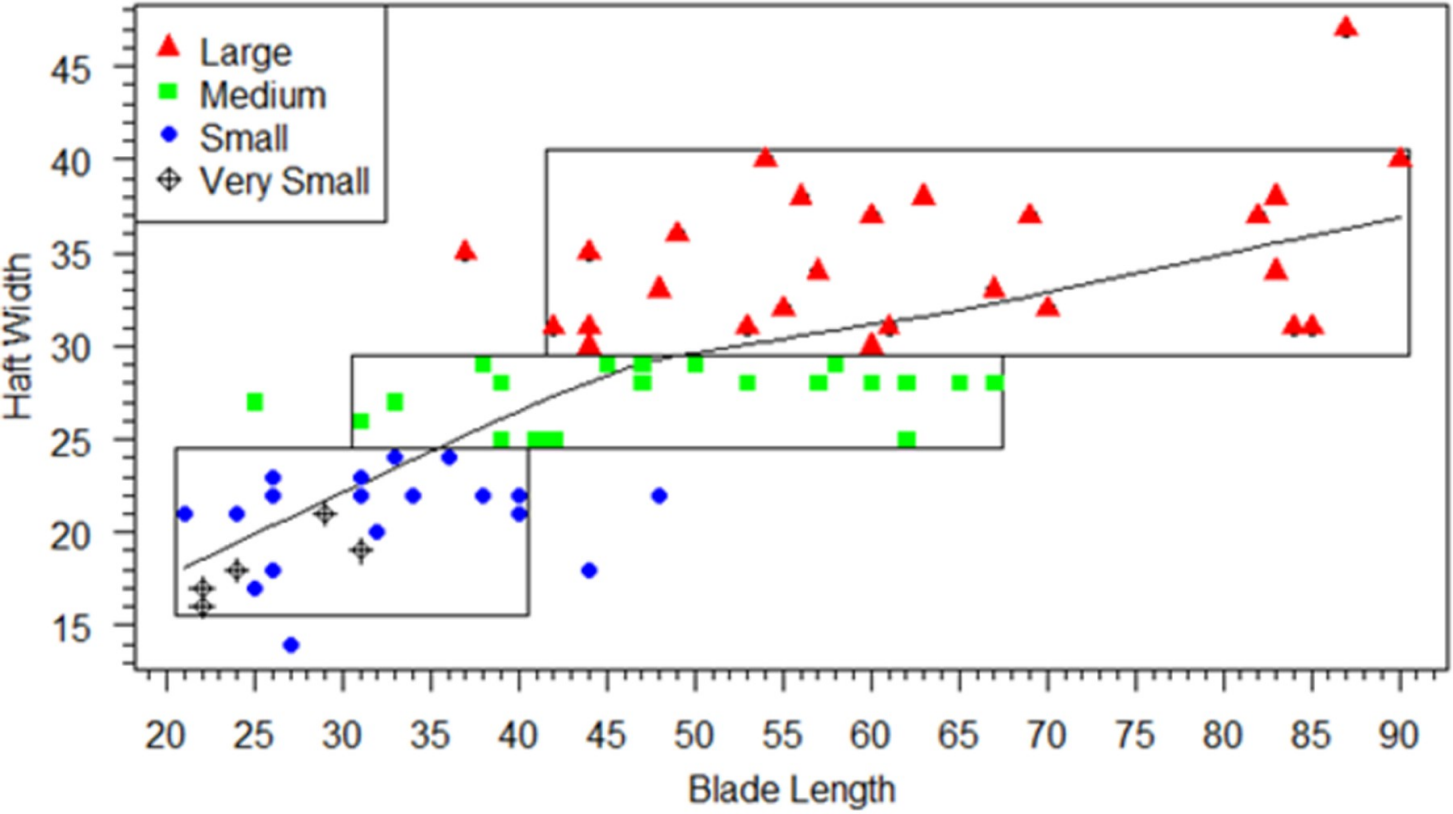
Scatterplot of haft-width by blade-length. LOWESS line and size classes indicated. Boxes enclose the concentrations of points in each haft-width size-category.

**Fig 8:**
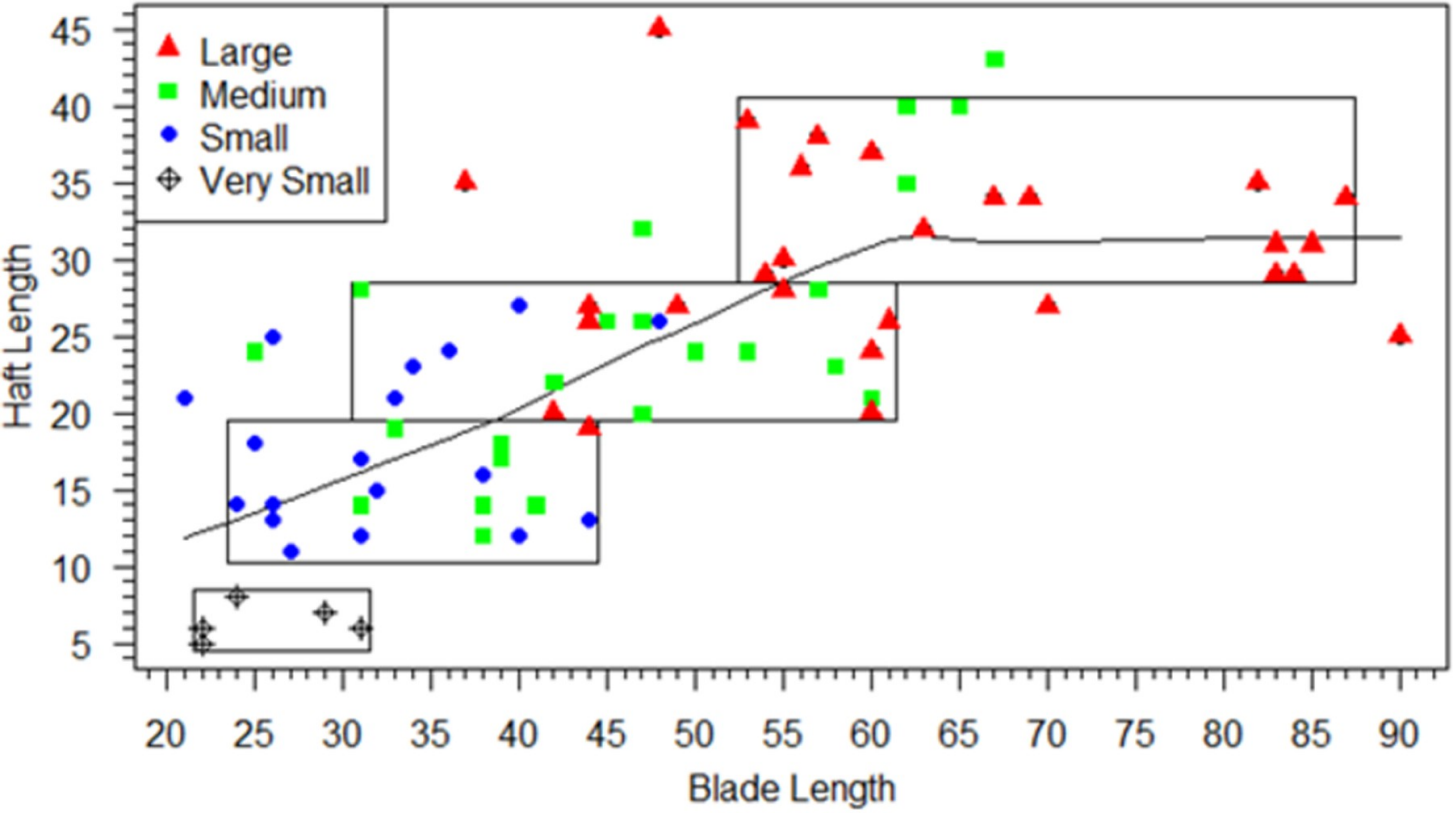
Scatterplot of haft-length by blade-length. LOWESS line and size classes indicated. Boxes enclose the concentrations of points in four heuristic haft-length size-categories.

We start with the assumption that in the integrated Clovis point design, hafts must be of sufficient size and shape to hold blades safely and effectively for the tool to function. Our use of technological criteria to define blade/haft junctions allow us to explore these haft design criteria. Neither module is independent of the other in the proper functioning of the tool, but each has different functional limitations. Hafts that are too short or narrow might allow the tool to slip or come loose in the handle rendering its use unsafe or ineffective. This assumption does not depend on how the point was attached to the handle or its shape, which for Clovis points is unknown and subject to debate (e.g., [22, 26, 42, 85]). Regardless of how hafts were hafted, the strong integration of point hafts and blades indicates that proper functioning of the Clovis point required the haft and blade to be about the same width. Additionally, configurations of hafts and blades were intended to maximize useable blade-lengths because blades did the work and hafts were designed to hold the blades securely in the correct position. We can examine the haft and blade modules design constraints by comparing linear dimensions of the modules in three bivariate plots of haft length and width and blade width (Figs 6–8).

To facilitate analysis and visualization of haft functional constraints, four heuristic haft-width size classes were designated in the scatterplot of haft-width to haft-length (Fig 6), with haft-width as the discriminating size-class dimension. Eighteen small category points (solid blue circles) have haft-widths between 17 and 24 mm; 23 medium category points (green squares) ranged from 25 to 29 mm, and 28 large points (solid red triangles) ranged from 30–40 mm. Five small points (three from Blackwater Draw and two from Lehner) were included in a separate “very small” category (open circles with crosses) because they have significantly shorter haft-lengths ranging from 5 to 8 mm. Boxes enclose the main concentrations of each category. Whether these size-classes represent intention at manufacture or just capture variation in a continuum of resharpening and reworking is unknown. Nonetheless, the classes are heuristics for illustrating the functional constraints of covarying haft and blade linear measurements. The dimension ranges for haft, blade, and maximum lengths organized by haft-width size-classes are listed in Table 2. We agree with Kilby [5] that at least some cache points had special uses, which can be inferred from Figs 4, 5 and the linear data in S1 Table, so we limit our analyses of functional constraints to the 74 non-cache points.

### Non-cache point functional constraints

Although hafts serve a supporting role in hafted-tool functionality, Rots [130, 131] has shown they hold valuable information on tool function. In the following analysis, we consider blades but focus on haft shapes (Fig 3B, LMs 1–4, 6–13). Within the haft, shape is allometric with haft-length but not haft-width (S2 Table 4 in S2 Table), meaning that of the two heuristic size-classes, haft shapes are primarily affected by changes in haft-lengths but not haft-widths. This inference is confirmed by the coefficient of determination (r^2^) results where haft-length contributes to about 9% of the variation but haft-width contributes about 1%, essentially no contribution.

The scatterplot of haft-length to haft-width (Fig 6; dot color and shapes are the same in Figs 7 and 8) illustrates that haft-lengths are variable with high overlap between heuristic haft-width size-classes, supporting the earlier analyses showing haft-lengths capture most of the haft shape variation (S2 Table 4 in S2 Table). Fig 7, a plot of haft-widths and blade-lengths, clarifies functional limits of hafts. For the small class, blades were no longer than ∼48 mm and rarely longer than ∼40 mm, from which we infer small hafts could not adequately hold blades larger than 48 mm. The medium-width hafts held blades up to ∼67 mm, and large-width hafts could hold blades up to ∼90 mm in length. Haft-length also had functional constraints. Fig 8 plots haft-length against blade-length, and the majority of points in the four heuristic haft-length size classes are circumscribed by boxes. In the very small category, haft lengths between 5 and 8 mm could only hold blades up to 31 mm in length. Usually, haft-lengths must have been at least ∼10 mm, and small category haft-lengths (10–19 mm) could hold blades no more than ∼48 mm in length. Medium-length hafts typically held blades no longer than 61 mm. Hafts ∼26 mm and longer were needed to hold larger blades between ∼62 to ∼90 mm long.

LOWESS (LOcally WEighted Scatterplot Smoothing) lines are plotted in Figs 6–8 and provide some insight into how a Clovis point might have been resharpened, regardless of its starting size. LOWESS lines, which are nonparametric regressions that express local relationships (i.e., without outliers) with smooth lines in a scatterplot, are often used in exploratory data analysis for time series data. We can think of the bivariate plots as capturing either several initial sizes or the changes through time in several dimensions of Clovis points from manufacture to discard. In the latter case, the LOWESS line approximates the dimensional changes experienced by individual Clovis points that transit most of the resharpening trajectory. A typical larger point might have been made with a 90 mm blade and a haft that was ∼32 mm long (Fig 8) and ∼37 mm wide (Fig 7). The blade was resharpened or repaired by removing flakes around its edges, thereby narrowing and shortening it. Earlier we proposed that when large Clovis point blades were resharpened, point hafts could have been shortened to maintain a long blade until reaching a length where the shortened haft could no longer adequately hold the blade in place. We also know from the strong integration results, the width of the haft was generally the same as the blade, so hafts should have been narrowed as the blades were resharpened.

**Fig 9:**
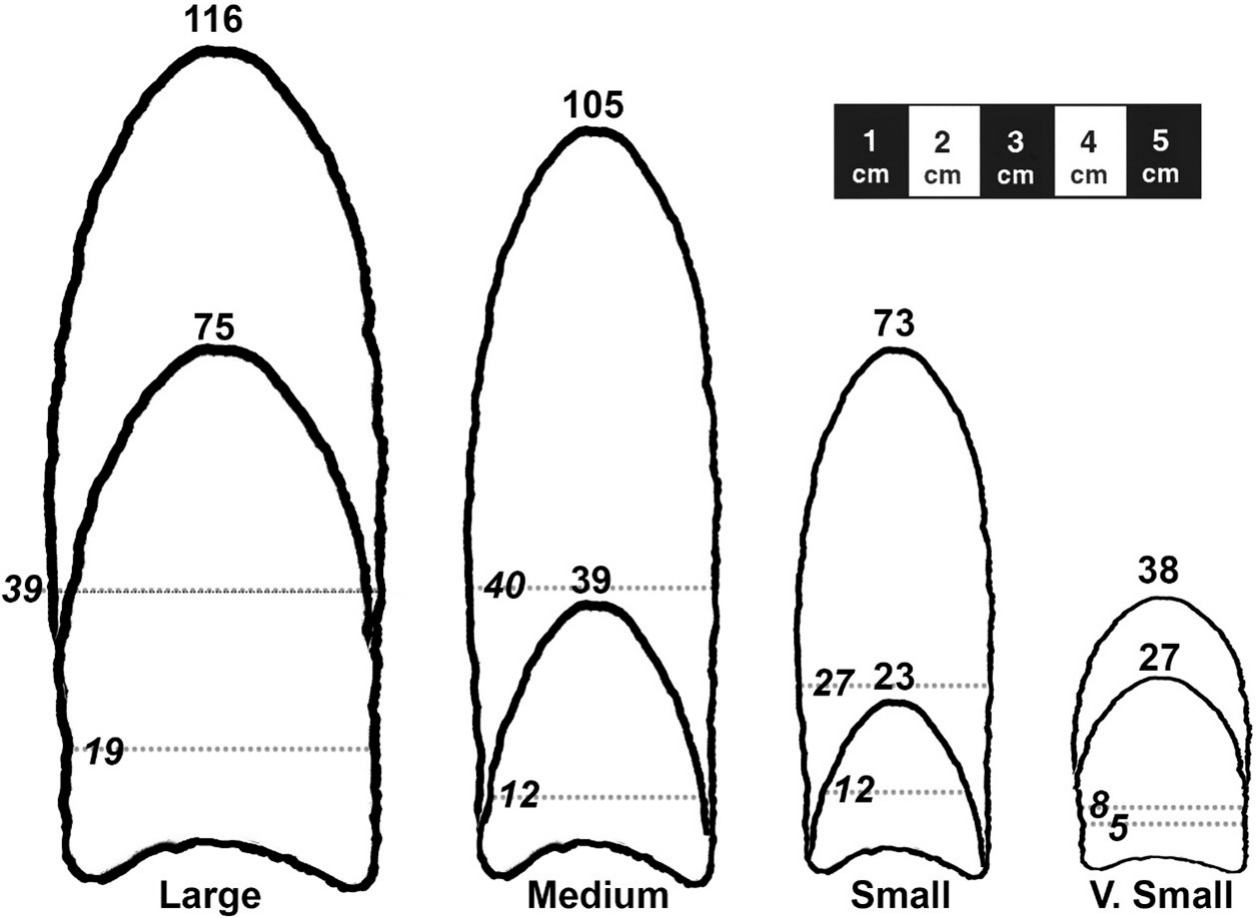
Longest and shortest points in each non-cache haft-width size-class. Maximum lengths of points are listed; haft-blade junctures indicated by dotted lines and italicized haft-lengths. Width variation in each size-class is not illustrated.

The LOWESS lines in Figs 7 and 8 indicate that as the blade was resharpened, the haft-length stayed the same until the blade reached ∼60 mm, but the haft-width gradually narrowed until it reached ∼31 mm. At ∼60 mm blade-length, the haft-length reached an inflection point and began to shorten at a constant rate until it reached about 12 mm. Haft shortening would be necessary to maximize usable blade length. Otherwise, usable stone that could be used in the tool’s function would be “wasted” in the haft. The haft-length shortened at half the rate as the blade (∼1 mm for every 2 mm of blade length loss). The haft-width narrowed ∼1 mm for every 6 mm of blade length loss until the blade reached 47 mm, at which time the rate of haft narrowing increased to ∼1 mm for every 2 mm of blade length loss. In sum, as predicted, haft-width started narrowing as soon as blades shortened (and presumably narrowed) to maintain the general haft-width/blade-width parity. In contrast, haft-length was steady until blade-length reached about 60 mm and then started to shorten. We interpret the 60 mm as generally the longest blade-length at which shorter hafts could safely hold the blade, and it also marked the maximum blade-length for the medium-size haft-lengths (Fig 8). But there certainly were exceptions to this general trend as shown by points outside the boxes. At blade-length ∼47 mm, haft-width also reached an inflection point at which its rate of narrowing increased until about 18 mm (Fig 7). Haft narrowing became more pronounced at an inflection point of ∼ 30 mm, when blade-length was about ∼45 mm. Hafts narrowed and shortened at about the same rate when hafts reached ∼30 in length and 32 mm in width (Fig 7). Fig 9 summarizes the haft and blade size variation by presenting the largest and smallest points in each haft-width size-class and illustrates the overlap in haft-length and blade-length in each class.

## Conclusion

Why does it matter that Clovis points are allometric and their blades and hafts are modular and integrated? First, we get a more accurate understanding of Clovis point design and life-history, and it is satisfying to demonstrate that Clovis points follow a general allometry pattern like the rest of North American points, with blade shapes more variable than haft shapes. Second, the high integration of haft and blade modules puts overall Clovis point shape in clearer context. We can better understand how Clovis point-makers and users thought about safety, effectiveness, and efficiency and how those concerns were manifested in the points. Third, if one is interested in using Clovis point shapes to infer spatial or temporal social relationships, the fact that blade-shape variation contributes to nearly 35% of total point shape variation must be taken into consideration when using entire point shapes for such analyses [92, 93, 132].

Of course, allometry should be neither assumed nor assumed away. We explicitly tested for allometry in a sample of Clovis points using LGM methods designed in part for that purpose. Results corroborate the general consensus on allometry’s relationship to resharpening. Previous LGM studies that failed to detect allometry in Paleoindian tools either are interesting exceptions to a general pattern recognized by most lithic analysts or limited by their research design and methodology. To resolve those possibilities, the datasets should be reanalyzed but only after defining stem and blade modules *independently of their landmarking or similar coding schemes*. Then original design and the possibility of subsequent allometry can be distinguished as sources of morphometric variation in Paleoindian points. Only if allometry is sought can its absence be demonstrated rather than assumed. The sometimes far-reaching implications drawn (e.g., tempo and mode of prehistoric colonization, the rates, patterns and causes of type diversification, standardization and its relationship to functional specificity, scaling laws among types) from studies that do not explicitly test for and therefore control for allometry also can be evaluated.

Finally, this work demonstrates the importance of considered LM placement. Most LGM analyses of LMs use many more semilandmarks than regular LMs, which may significantly affect statistical results and interpretations [93]. Here we show that LM placement criteria should capture the technological aspect of the artifact that is being studied. We were interested in the relationships between the blades and hafts, so defining those precisely with LMs was critical, but that LM pattern will not be appropriate for all artifact shape analyses [e.g., 55]. If LM placement is not technologically justifiable, the results will be suspect.

## Supporting information

S1 Fig Site locations. Dent Area Sites include Dent, Drake, Fox, Greeley, and Keresy Gravel Pit. San Pedro Valley Sites include Murray Springs, Lehner, Escapule, Leikem, Naco, and Shaldack. Base map made with Natural Earth public domain map data.
(PDF)

S1 File RMarkdown file. This file includes all the code for running the analyses and creating most of the figures in the Clovis Allometry article. The file also includes some analyses and figures that were not included in the publication.
(DOCX)

S2 File (ZIP)

S1 Table Details about each of the points used in the analyses. Image Name, ID (tps file ID), Haft Width (HaftW), Haft Length (HaftL), Blade Length (BladeL), Maximum Length (MaxL), Cache/Non-cache, Class (Haft width size category), and Haft Width/Maximum Length ratio (SWML).
(DOCX)

S2 Table (ZIP)
