# Supplementary material for: Clovis point allometry, modularity, and integration: Exploring shape variation due to tool use with landmark-based geometric morphometrics
Source: PLoS One. 2023 Aug 16;18(8):e0289489. doi: 10.1371/journal.pone.0289489 (PMC10431674; doi:10.1371/journal.pone.0289489)
Supplement: S1 Fig — Dent Area Sites include Dent, Drake, Fox, Greeley, and Keresy Gravel Pit. San Pedro Valley Sites include Murray Springs, Lehner, Escapule, Leikem, Naco, and Shaldack. Base map made with Natural Earth public domain map data. (PDF) [file pone.0289489.s001.pdf]

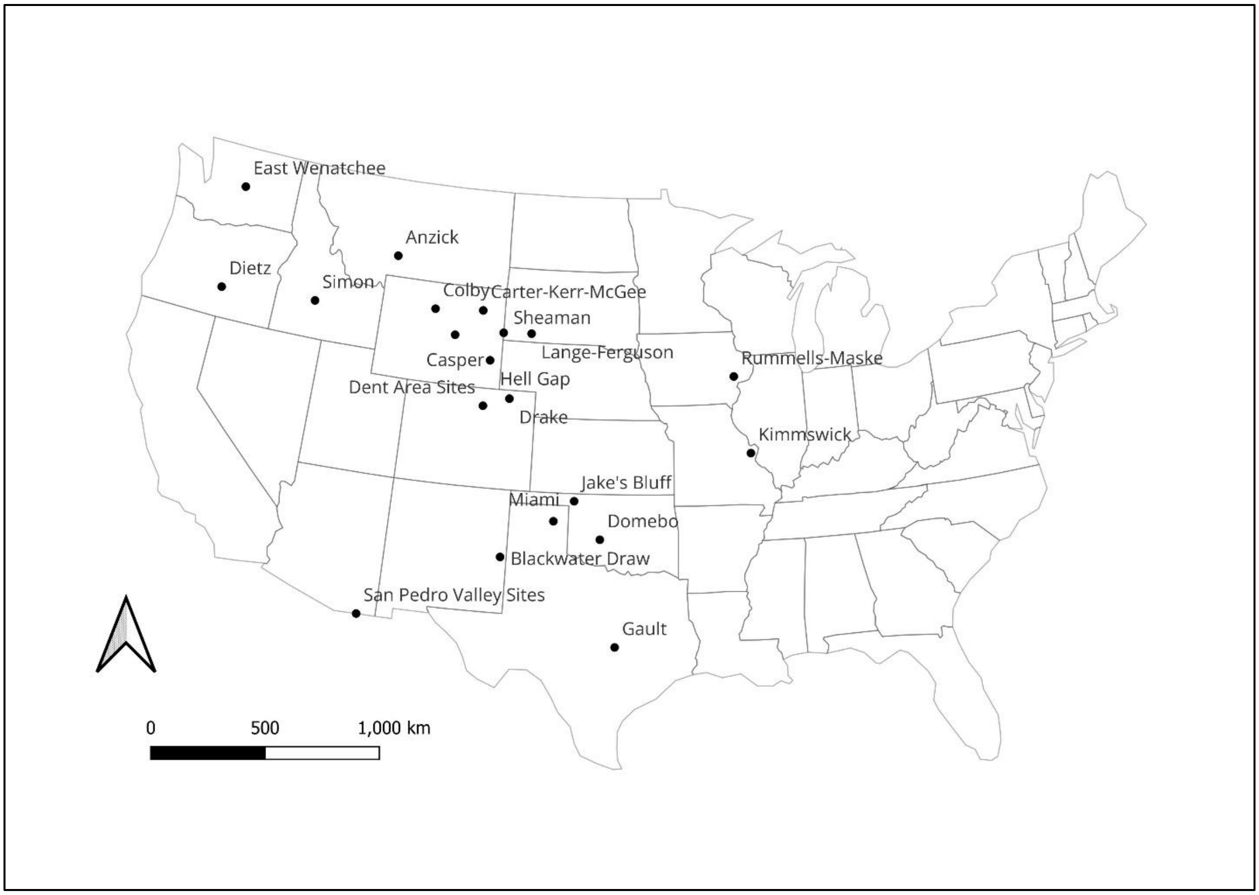

Figure S1: Site locations. Dent Area Sites include Dent, Drake, Fox, Greeley, and Keresy Gravel Pit. San Pedro Valley Sites include Murray Springs, Lehner, Escapule, Leikem, Naco, and Shaldack. Base map made with Natural Earth public domain map data.
