# Supplementary material for: Clovis point allometry, modularity, and integration: Exploring shape variation due to tool use with landmark-based geometric morphometrics
Source: PLoS One. 2023 Aug 16;18(8):e0289489. doi: 10.1371/journal.pone.0289489 (PMC10431674; doi:10.1371/journal.pone.0289489)
Supplement: S1 File — This file includes all the code for running the analyses and creating most of the figures in the Clovis Allometry article. The file also includes some analyses and figures that were not included in the publication. (DOCX) [file pone.0289489.s002.docx]

Clovis Point Allometry

David K. Thulman

1/25/2023

This file includes all the code for running the analyses and creating most of the figures in the Clovis Allometry article. The file also includes some analyses and figures that were not included in the publication. We’ve included detailed descriptions for what is going on in the code, but if you have quesitons, please contact the primary author. Some things to keep in mind:

1. The analyses are created with 3 files: CA23a.tps, which is a tps file created in tpsDig2; CA Data1.csv, which is a csv file with measurement and classification data; and EMSL.csv, which is a csv file that creates linking lines between landmarks for illustration.
2. Some abbreviations should help you navigate the code:

CA=Clovis Allometry (or CAP=Clovis Allometry Project). Numbers following CA indicate number of LMs. For example, “CA11Blade” means Clovis Allometry 11 LMs Blade-shape.

GPA=Generalized Procrustes Analysis.

GDF=geomorph dataframe.

CS=Centroid Size.

lnCS=log natural centroid size, followed by E (entire), B (blade), or H (haft).

O=Other, which is the designation given to non-cache points.

C=Cache.

1. Some functions are disabled by placing a # in front of the line of code. The picknplot and define.links functions require interactive actions, which interfere with the Rmd file. Refer to the geomorph instructions for guidance.

For those unfamiliar with R, there are often several ways to do the same thing in data manipulation, plotting, or statistical analysis, and sometimes this code uses different instructions to achieve the same result.

The analyses require three R packages

if (!require(pacman)) install.packages("pacman")

## Loading required package: pacman

pacman::p_load(tidyverse,
 Hmisc,
 geomorph,
 update = FALSE)

Set the working directory. This should be set to the directory in which you have loaded this Rmd file and the 3 other files listed in the introduction.

#setwd("D:/Documents/Analysis V/Allometry Clovis")

First, create a GDF file that contains the Entire point dataset. This takes 6 steps. We start by loading the CA23a.tps file for all the Clovis points used in the analyses, 2. perform a Generalized Procrustes Analysis (GPA) on the tps data, and save the results as an object named Z.gpa. The GPA calculates the centroid size, which is included in this object. 3. The CA Data1.csv data are loaded and an object named “groups” is created. 4. a geomorph dataframe (GDF) is created from the Z.gpa object, and 5. the groups object is appended to it. 6. The natural log of the entire-point CS is created and added to the CA23.gdf object in a new list called lnCSE.

CAP23<-readland.tps("CA23a.tps", specID = "ID") # 1. Reads in the 23LM tps data and creates a new object CAP23

##
## No curves detected; all points appear to be fixed landmarks.

Z.gpa<-gpagen(CAP23, print.progress = FALSE) # 2. Generalized Procrustes Analysis preformed and resulting object named as Z.gpa
groups<-read.csv("CA Data1.csv",header=TRUE,stringsAsFactors = TRUE) # 3. Reads in the classifier data as factors
CA23.gdf <- geomorph.data.frame(Z.gpa) # 4. Creates a dataframe from the Z.gpa output named CA23.gdf
CA23.gdf <- append(CA23.gdf, groups) # 5. Appends groups factors to the dataframe created from Z.gpa, keeps same name
CA23.gdf$lnCSE <- log(CA23.gdf$Csize) # 6. Creates new column in the dataframe with natural log of entire centroid Size

Now we create 2 additonal GDF dataframes for the blade and haft modules. We want to keep the LM configurations of these modules from the GPA of the entire shape, so the procedure is more complex than if we treated the modules as independent shapes. We start by 1. creating a copy of the CA23.gdf dataframe, which will be a placeholder we will modify to include only the haft-shape coordinates and centroid sizes. 2. Next, we create an object that lists the blade LMs we will delete to leave the haft shape. 3. The omit.blade object is subtracted from the coordinates in the GDF file and a new object (HHH) is created. 4. These 12 coordinates for each Clovis point haft replace the entire shape 23LM coordinates.

CAHaft.gdf <- CA23.gdf # 1. Creates a copy of the 23Lm configuration
omit.blade <- c(5,14:23) # 2. Creates object of LMs (named "omit.blade") to delete the blade LMs from the 23LM tps file
HHH <- CAHaft.gdf$coords [-omit.blade, ,] # 3. SSS is a placeholder object that now includes the Haft-shape coordinates
CAHaft.gdf$coords <- HHH # 4. SSS replaces the CAHaft.gdf coordinates

Now we want to replace the 23LM CS with the Haft CS and include a list of the ln of that CS. First, we need to create the Haft CS, which is done by subtracting the 11 LM blade configuration from the 23 LM tps file using the omit.blade object created earlier. This is different from what was done above because we are only interested in the Haft CS, which is measured from the pre-GPA LM configurations and is not dependent on how the LMs are distributed after GPA. 1. The new CA12Haft object with 12 LMs is 2. run through GPA, and the results 3. converted to a GDF dataframe. 4. The CS is saved as an HCS object, 5. which replaces the CS data in the CAHaft.gdf dataframe. The last step 6. is to add the natural log of the CS (lnCSS) as a new list. 7. Finally, the results are checked by plotting all 100 Clovis point LMs and their mean LM configuration.

CA12Haft <- CAP23 [-omit.blade, ,] # 1. Subtracts 11 blade LMs from the 23LM tps file to create a Haft shape tps file with 12 LMs
CAHaft.gpa <- gpagen(CA12Haft, print.progress = FALSE) # 2. Generalized Procrustes analysis for Haft shape in new object CAHaft.gpa
HaftCS.gdf <- geomorph.data.frame(CAHaft.gpa) # 3. Creates a Haft geomorph dataframe, solely for extracting the Haft CS
HCS <- HaftCS.gdf$Csize # 4. Copies Haft CS to an interim object
CAHaft.gdf$Csize <- HCS # 5. Interim object replaces Entire CS with Haft CS
CAHaft.gdf$lnHCS <- log(CAHaft.gdf$Csize) # 6. Creates new list with natural log of Haft CS
plotAllSpecimens(CAHaft.gdf$coords, mean=TRUE, plot.param = list(pt.cex=.6))


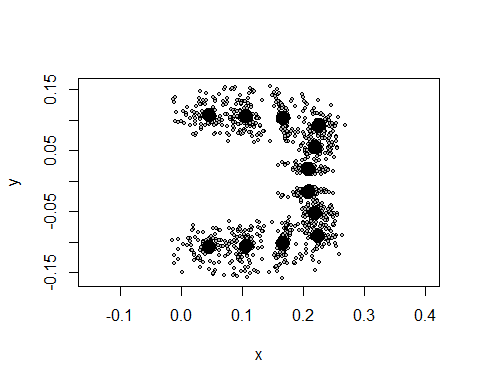


The same steps are followed to create the blade GDF.

CABlade.gdf <- CA23.gdf # Creates a copy of the 23Lm configuration, but we need to change the number of LMs
omit.Haft <- c(1:4,6:13)# creates vector of Haft LMs (named "omit.Haft") to delete the Haft LMs from the 23LM tps shape
BBB <- CABlade.gdf$coords [-omit.Haft, ,] # BBB is a placeholder object with only the Blade LMs
CABlade.gdf$coords <- BBB # BBB replaces the 23 LM coordinates
#Now we need to create Blade CS and replace the Entire CS in CA11Blade.gdf
CA11Blade <- CAP23 [-omit.Haft, ,] # Subtracts 12 haft LMs from the 23LM tps file to create a Blade shape tps file with 11 LMs
CABlade.gpa <- gpagen(CA11Blade, print.progress = FALSE) # Generalized Procrustes Analysis for Blade shape in new object CABlade.gpa
BladeCS.gdf <- geomorph.data.frame(CABlade.gpa) # Creates a Blade geomorph dataframe, solely for extracting the CS
BCS <- BladeCS.gdf$Csize # Copies Blade CS to an interim object
CABlade.gdf$Csize <- BCS # Interim object replaces Entire-shape CS with Blade CS
CABlade.gdf$lnCSB <- log(CABlade.gdf$Csize) # Creates new column with natural log of Blade centroid size
plotAllSpecimens(CABlade.gdf$coords, mean=TRUE, plot.param = list(pt.cex=.6)) # Checks the new Blade LM distribution


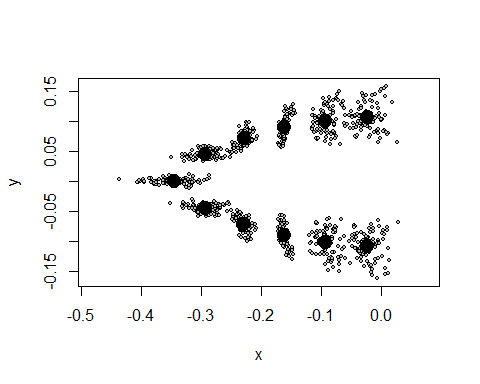


We add the Haft and Blade lnCSs to the Entire-point dataframe for later analyses.

CA23.gdf$lnCSS <- CAHaft.gdf$lnCSH # Creates new column with natural log of Haft CS
CA23.gdf$lnCSB <- CABlade.gdf$lnCSB # Creates new column with natural log of Blade CS

2 plots are created. First, mean shape of the entire 23LM configuration and a links file that connects adjacent LMs with lines. This is Figure 2A. The second plot is of the LMs of all the Clovis points around the mean LM locations. This is Figure 2B. 1. For 2A, a mean shape is extracted from the 23LM configuration. 2. A plotting configuration is set. 3. The interactive define.links function is disabled. 4. The EMSL.csv file was previously created by define.links is read in and 5. appended to the CA23.gdf dataframe. 6. The mean shape (MS) with the links is plotted (Figure 2A). 7. Figure 2B is plotted.

MS <- mshape(CA23.gdf$coords) # 1. Creates mean shape of 23LM shape data and labels LMs
par(mfcol = c(1,1)) # 2. Sets single-plot option
#EMSL<- define.links(MS) # 3. Create a links file named EMSL. Interactive function disabled
EMSL <- read.csv("EMSL.csv") # 4. Reads the links file in an object EMSL
CA23.gdf<-append(CA23.gdf, EMSL) # 5. Appends the links file EMSL to the dataframe
plotAllSpecimens(CA23.gdf$coords, mean=TRUE, plot.param = list(pt.cex=.6), links=EMSL)# 6. Distibution of LMs from all specimens with the links file [Figure 2B]


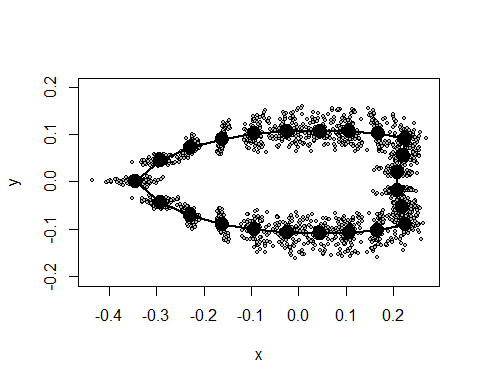


plot(MS, links=EMSL) # 7. Plots mean shape with LM links [Figure 2A]


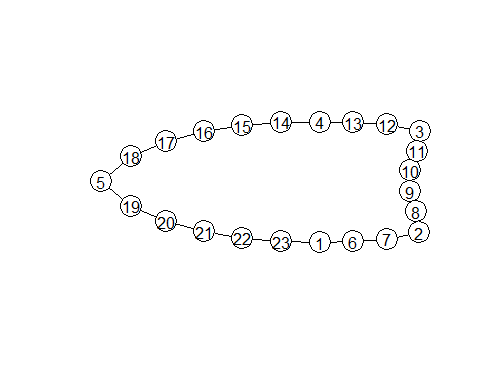


This is the first analysis. It uses the procD.lm function, which uses GPA data to quantify the relative amount of shape variation. The procD.lm function is used in these analyses to produce ANOVA and regressions using various factors. The function produces p-values through the package RRPP, which uses residual randomization. Here, we analyze the allometry signal in the 23LM coordinates (coords) against natural log of CS - log(Csize) - and create an object: CA23CSE. The summary function calls the ANOVA results, which are in Supplemental Table 2.

CA23CSE <- procD.lm(coords~ log(Csize), data=CA23.gdf, iter=10000, RRPP=TRUE, print.progress = FALSE) # Creates object for regression of coords on log Centroid size
summary(CA23CSE) # ANOVA results in [Supplemental Table 2]

##
## Analysis of Variance, using Residual Randomization
## Permutation procedure: Randomization of null model residuals
## Number of permutations: 10001
## Estimation method: Ordinary Least Squares
## Sums of Squares and Cross-products: Type I
## Effect sizes (Z) based on F distributions
##
## Df SS MS Rsq F Z Pr(>F)
## log(Csize) 1 0.47478 0.47478 0.36038 55.217 6.3035 9.999e-05 ***
## Residuals 98 0.84265 0.00860 0.63962
## Total 99 1.31743
## ---
## Signif. codes: 0 '***' 0.001 '**' 0.01 '*' 0.05 '.' 0.1 ' ' 1
##
## Call: procD.lm(f1 = coords ~ log(Csize), iter = 10000, RRPP = TRUE,
## data = CA23.gdf, print.progress = FALSE)

The following plot of entire points data is not published but shows the preceding allometry results with cache and non-cache points designated by colors and shapes. The picknplot.shape function, which creates the wireframe graphs of individual points, is disabled.

color = rep(NA, length=length(CA23.gdf$Cache)) #colors cache and non-cache dots in plot
color[which(CA23.gdf$Cache=="Cache")] = "black"
color[which(CA23.gdf$Cache=="Other")] = "red"
#color #check to determine color designations are correct - DISABLED
shape = rep(NA, length=length(CA23.gdf$Cache)) #shapes for cache and non-cache dots in plot
shape[which(CA23.gdf$Cache=="Other")] = 1 # Open circles
shape[which(CA23.gdf$Cache=="Cache")] = 17 # Solid triangles
plotAllometry(CA23CSE, size=CA23.gdf$Csize, method = "RegScore", col=color, pch=shape, bg=CA23.gdf$Cache, xlab="Ln Entire Centroid Size") # Plots the Regression Scores against ln Centroid Size


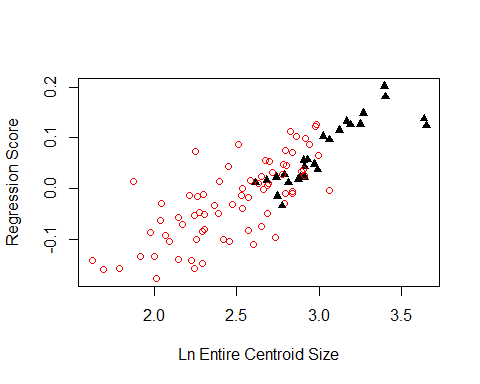


The same again for the Haft. The ANOVA results are in Supplemental Table 2.

CAHaftCS <- procD.lm(coords~ log(Csize), data=CAHaft.gdf, iter=10000, RRPP=TRUE, print.progress = FALSE) #names regression of coords on log Centroid size
summary(CAHaftCS) # Calls ANOVA details in [Supplemental Table 2]

##
## Analysis of Variance, using Residual Randomization
## Permutation procedure: Randomization of null model residuals
## Number of permutations: 10001
## Estimation method: Ordinary Least Squares
## Sums of Squares and Cross-products: Type I
## Effect sizes (Z) based on F distributions
##
## Df SS MS Rsq F Z Pr(>F)
## log(Csize) 1 0.16521 0.165214 0.21565 26.945 5.0369 9.999e-05 ***
## Residuals 98 0.60089 0.006132 0.78435
## Total 99 0.76611
## ---
## Signif. codes: 0 '***' 0.001 '**' 0.01 '*' 0.05 '.' 0.1 ' ' 1
##
## Call: procD.lm(f1 = coords ~ log(Csize), iter = 10000, RRPP = TRUE,
## data = CAHaft.gdf, print.progress = FALSE)

An unpublished plot for Haft-shapes, like the preceding with entire-shapes plot.

color = rep(NA, length=length(CAHaft.gdf$Cache)) #colors cache and non-cache dots in plot
color[which(CAHaft.gdf$Cache=="Cache")] = "black"
color[which(CAHaft.gdf$Cache=="Other")] = "red"
#color #check to determine color designations are correct - DISABLED
shape = rep(NA, length=length(CAHaft.gdf$Cache)) #colors cache and non-cache dots in plot
shape[which(CAHaft.gdf$Cache=="Other")] = 1 # Open circles
shape[which(CAHaft.gdf$Cache=="Cache")] = 17 # Solid triangles
plotAllometry(CAHaftCS, size=CAHaft.gdf$lnHCS, logsz=FALSE, method = "RegScore", col=color, pch=shape, bg=CAHaft.gdf$Cache, xlab="Ln Haft Centroid Size") # Plots the Regression Scores against ln Centroid Size


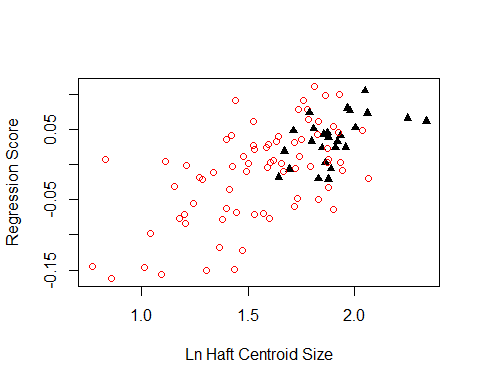


#CAHaftAllometry <- plotAllometry(CAHaftCS, size=CAHaft.gdf$lnCSS, logsz=FALSE, method = "RegScore", col=color, pch=shape, bg=CAHaft.gdf$Cache, xlab="Ln Haft Centroid Size") # creates object for picknplot function
# picknplot.shape(CAHaftAllometry) #creates the small inserts, interaction function disabled

Now the blade-shapes

CABladeCS <- procD.lm(coords~ log(Csize), data=CABlade.gdf, iter=10000, RRPP=TRUE, print.progress = FALSE) # names object of regression of Blade coords on log Centroid size
summary(CABladeCS) # ANOVA results in [Table 2]

##
## Analysis of Variance, using Residual Randomization
## Permutation procedure: Randomization of null model residuals
## Number of permutations: 10001
## Estimation method: Ordinary Least Squares
## Sums of Squares and Cross-products: Type I
## Effect sizes (Z) based on F distributions
##
## Df SS MS Rsq F Z Pr(>F)
## log(Csize) 1 0.22909 0.229093 0.41553 69.674 6.6332 9.999e-05 ***
## Residuals 98 0.32223 0.003288 0.58447
## Total 99 0.55132
## ---
## Signif. codes: 0 '***' 0.001 '**' 0.01 '*' 0.05 '.' 0.1 ' ' 1
##
## Call: procD.lm(f1 = coords ~ log(Csize), iter = 10000, RRPP = TRUE,
## data = CABlade.gdf, print.progress = FALSE)

And the unpublished plot of blade-shapes.

color = rep(NA, length=length(CABlade.gdf$Cache)) #colors cache and non-cache dots in plot
color[which(CABlade.gdf$Cache=="Cache")] = "black"
color[which(CABlade.gdf$Cache=="Other")] = "red"
#color #check to determine color designations are correct - DISABLED
shape = rep(NA, length=length(CABlade.gdf$Cache)) #colors cache and non-cache dots in plot
shape[which(CABlade.gdf$Cache=="Other")] = 1 # Open circles
shape[which(CABlade.gdf$Cache=="Cache")] = 17 # Solid triangles
plotAllometry(CABladeCS, size=CABlade.gdf$lnCSB, logsz=FALSE, method = "RegScore", col=color, pch=shape, bg=CABlade.gdf$Cache, xlab="Ln Blade Centroid Size") # Plots the Regression Scores against ln Centroid Size


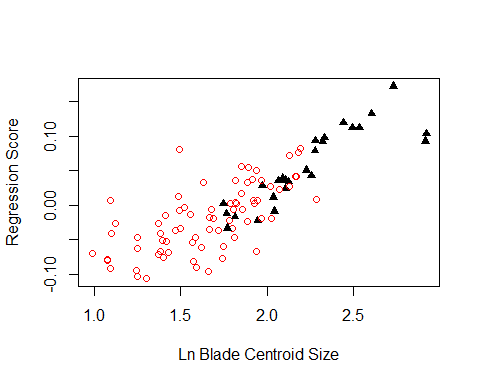


#CABladeAllometry <- plotAllometry(CABladeCS, size=CABlade.gdf$lnCSB, logsz=FALSE, method = "RegScore", col=color, pch=shape, bg=CABlade.gdf$Cache, xlab="Ln Blade Centroid Size") # creates object for picknplot function
# picknplot.shape(CAHaftAllometry) #creates the small inserts, interaction function disabled

Results of ANOVAs referred to in the text and reported in Supplemental Table 3 comparing cache and non-cache Entire, Blade, and Haft shapes. All are significantly different. First, the entire shapes.

fitE <- procD.lm(coords ~ Cache, data = CA23.gdf, print.progress = FALSE)
anova(fitE)

##
## Analysis of Variance, using Residual Randomization
## Permutation procedure: Randomization of null model residuals
## Number of permutations: 1000
## Estimation method: Ordinary Least Squares
## Sums of Squares and Cross-products: Type I
## Effect sizes (Z) based on F distributions
##
## Df SS MS Rsq F Z Pr(>F)
## Cache 1 0.1935 0.193502 0.14688 16.872 4.1294 0.001 **
## Residuals 98 1.1239 0.011469 0.85312
## Total 99 1.3174
## ---
## Signif. codes: 0 '***' 0.001 '**' 0.01 '*' 0.05 '.' 0.1 ' ' 1
##
## Call: procD.lm(f1 = coords ~ Cache, data = CA23.gdf, print.progress = FALSE)

Next, the Blade shapes.

fitB <- procD.lm(coords ~ Cache, data = CABlade.gdf, print.progress = FALSE)
anova(fitB)

##
## Analysis of Variance, using Residual Randomization
## Permutation procedure: Randomization of null model residuals
## Number of permutations: 1000
## Estimation method: Ordinary Least Squares
## Sums of Squares and Cross-products: Type I
## Effect sizes (Z) based on F distributions
##
## Df SS MS Rsq F Z Pr(>F)
## Cache 1 0.10398 0.103982 0.1886 22.779 4.366 0.001 **
## Residuals 98 0.44734 0.004565 0.8114
## Total 99 0.55132
## ---
## Signif. codes: 0 '***' 0.001 '**' 0.01 '*' 0.05 '.' 0.1 ' ' 1
##
## Call: procD.lm(f1 = coords ~ Cache, data = CABlade.gdf, print.progress = FALSE)

Next, the Haft shapes.

fitS <- procD.lm(coords ~ Cache, data = CAHaft.gdf, print.progress = FALSE)
anova(fitS)

##
## Analysis of Variance, using Residual Randomization
## Permutation procedure: Randomization of null model residuals
## Number of permutations: 1000
## Estimation method: Ordinary Least Squares
## Sums of Squares and Cross-products: Type I
## Effect sizes (Z) based on F distributions
##
## Df SS MS Rsq F Z Pr(>F)
## Cache 1 0.08952 0.089520 0.11685 12.966 3.7488 0.001 **
## Residuals 98 0.67659 0.006904 0.88315
## Total 99 0.76611
## ---
## Signif. codes: 0 '***' 0.001 '**' 0.01 '*' 0.05 '.' 0.1 ' ' 1
##
## Call: procD.lm(f1 = coords ~ Cache, data = CAHaft.gdf, print.progress = FALSE)

Figure 5 is a plot comparing the unique allometry of cache and non-cache points. Wire-frames are created and inserted into each showing shapes with the largest and smallest regression scores in each plot. The wireframes were created with picknplot.shape (disabled) and inserted in Photoshop.

fitX.common <- procD.lm(coords ~ log(Csize) + Cache,
 data = CA23.gdf, print.progress = FALSE) # Common allometry of cache and non-cache points
fitX.unique <- procD.lm(coords ~ log(Csize) * Cache,
 data = CA23.gdf, print.progress = FALSE) # Unique allometries of Cache, non-cache points
anova(fitX.common, fitX.unique, print.progress = FALSE) # Comparison of the common and unique allometries for significant differences (Supplemental Table 3)

##
## Analysis of Variance, using Residual Randomization
## Permutation procedure: Randomization of null model residuals
## Number of permutations: 1000
## Estimation method: Ordinary Least Squares
## Effect sizes (Z) based on F distributions
##
## ResDf Df RSS SS MS Rsq
## coords ~ log(Csize) + Cache (Null) 97 1 0.81046 0.000000
## coords ~ log(Csize) * Cache 96 1 0.73509 0.075369 0.075369 0.057209
## Total 99 1.31743
## F Z P Pr(>F)
## coords ~ log(Csize) + Cache (Null)
## coords ~ log(Csize) * Cache 9.8429 3.2247 0.002
## Total

fitXU<-plotAllometry(fitX.unique, size=CA23.gdf$Csize, logsz = TRUE, method = "RegScore", col=color, pch = shape,bg = CA23.gdf$Cache, xlab="Natural log Entire Centroid Size") # Figure 5


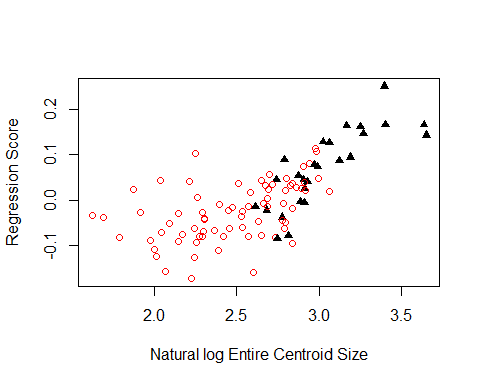


#picknplot.shape(fitXU) # Interactive function for Figure 5 disabled. Places shapes on plot

Integration test is performed on the GPA aligned coordinates file (Z.gpa). Plot not published.

CA23.inter <- integration.test(Z.gpa$coords[c(1:4,6:13),,], Z.gpa$coords[c(5,14:23),,], iter=999, print.progress = FALSE) # Divide entire shape into Haft and blade modules
summary(CA23.inter) # Provides r-PLS score

##
## Call:
## integration.test(A = Z.gpa$coords[c(1:4, 6:13), , ], A2 = Z.gpa$coords[c(5,
## 14:23), , ], iter = 999, print.progress = FALSE)
##
##
##
## r-PLS: 0.943
##
## Effect Size (Z): 7.5875
##
## P-value: 0.001
##
## Based on 1000 random permutations

plot(CA23.inter) # Plots PLS Blocks 1 and 2


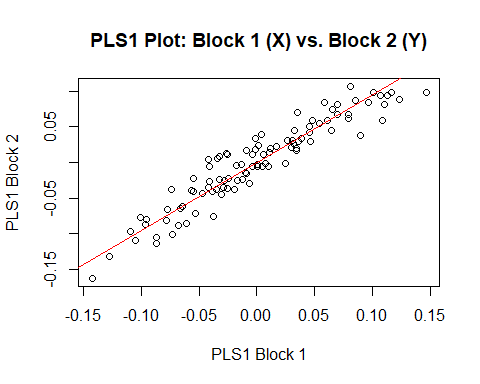


Modularity Testis performed on the GPA aligned coordinates file (Z.gpa). A histogram of the results is created and the abline of our results is plotted. Plot not published.

CA.gps <- c("A","A","A","A","B","A","A","A","A","A","A","A","A","B","B","B","B","B","B","B","B","B","B") # Creates Haft(A) and Blade(B) modules
modularity.test(Z.gpa$coords, CA.gps, iter=999, print.progress = FALSE) # Calculates CR statistic

##
## Call:
## modularity.test(A = Z.gpa$coords, partition.gp = CA.gps, iter = 999,
## print.progress = FALSE)
##
##
##
## CR: 1.0019
##
## P-value: 0.02
##
## Effect Size: -2.5663
##
## Based on 1000 random permutations

CA23.mod <- modularity.test(Z.gpa$coords, CA.gps, iter=999, print.progress = FALSE) # Creates object for histogram

hist(CA23.mod$random.CR,xlim = c(0.96, 1.09), xaxs="i",ylim = c(0,300), yaxs="i",xlab="CR Coefficient", main="CR Coefficient Distribution", col="gray") # Histogram showing CR results.
mean(CA23.mod$random.CR) # Finds the mean of the random.CR distribution

## [1] 1.049147

abline(v=1.049103, col="blue") # Abline line for the mean of the distribution
abline(v = 1.0018,col="red") # Abline line


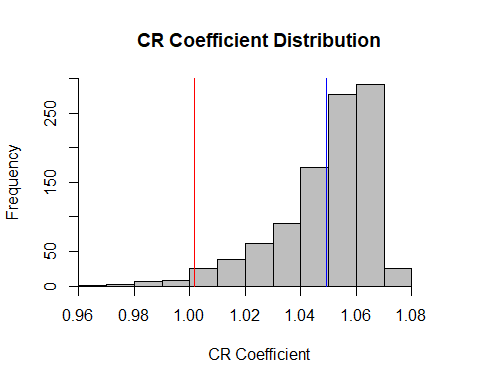


The following analyses concern the Clovis Hafts in non-cache points. Creating the dataframe is another multi-step process that requires deleting cache points and then the blade LMs. Again, we want to keep the original Haft GPA distributions using only non-cache points. 1. The first task is to remove the cache points from the 23LM tps file, creating a new CAP23O object (“Clovis Allometry Project 23LMs Other than cache points”) and (2) from the groups object, creating a new object, groupsO. 3. run the GPA function on CAP23O and create a new placeholder object OO.gpa. 4. Create the geomorph dataframe from the OO.gpa object created earlier. 5. Use omit.blade to create the Haft-shape LM configuration. 6. Replace the 23 LMs with the 12 Haft LMs. 7. Append the CA23O.gdf and groupsO objects into a new CAHaftO.gdf dataframe. 8. Plot all the specimens as a check.

CAP23O <- CAP23[,,-c(25:36,40:46,49:53,91,92)] # 1. Remove cache points from the 23LM tps file and creating a new "Clovis Allometry Project 23 Other" object.
groupsO <- groups[!(groups$Cache=="Cache"),] # 2. Remove cache points from the "groups" file loaded earlier.
OO.gpa<-gpagen(CAP23O, print.progress = FALSE) # 3. GPA on the non-cache tps file
CA23O.gdf <- geomorph.data.frame(OO.gpa) # 4. Create a geomorph dataframe from the GPA object
SSS <- CA23O.gdf$coords [-omit.blade, ,] # 5. Use the earlier "omit.blade" file to delete blade LMs and create a Haft-shape set of coordinates
CA23O.gdf$coords <- SSS # 6. Replace the 23LMs with the 12 Haft LMs
CAHaftO.gdf <- append(CA23O.gdf, groupsO) # 7. Append the new GDF with the new "groupsO" object
plotAllSpecimens(CAHaftO.gdf$coords, mean=TRUE, plot.param = list(pt.cex=.6)) # 8. Check to ensure the proper configuration and number of specimens is present


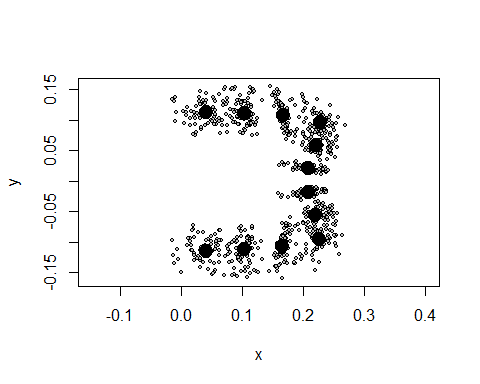


ANOVA results are created for Supplemental Table 3 using the Haft coordinates by the size-classes, and ln of the linear dimensions of Haft-Width and Haft-Length.

fitHO <- procD.lm(coords ~ Class, data = CAHaftO.gdf, print.progress = FALSE) # Comparison of Haft shape by size-class
anova(fitHO) # ANOVA Results for Haft size-classes (Supplemental table 4)

##
## Analysis of Variance, using Residual Randomization
## Permutation procedure: Randomization of null model residuals
## Number of permutations: 1000
## Estimation method: Ordinary Least Squares
## Sums of Squares and Cross-products: Type I
## Effect sizes (Z) based on F distributions
##
## Df SS MS Rsq F Z Pr(>F)
## Class 3 0.02475 0.0082484 0.04586 1.1214 0.39744 0.354
## Residuals 70 0.51489 0.0073556 0.95414
## Total 73 0.53964
##
## Call: procD.lm(f1 = coords ~ Class, data = CAHaftO.gdf, print.progress = FALSE)

Next, the Haft Shape ~ Haft Width is tested.

fitOHW <- procD.lm(coords~ log(HaftW), data=CAHaftO.gdf, iter=999, RRPP=TRUE, print.progress = FALSE) # Regression of Haft shape on log normal Haft width
anova(fitOHW)# ANOVA Results (Supplemental table 4) for Haft width

##
## Analysis of Variance, using Residual Randomization
## Permutation procedure: Randomization of null model residuals
## Number of permutations: 1000
## Estimation method: Ordinary Least Squares
## Sums of Squares and Cross-products: Type I
## Effect sizes (Z) based on F distributions
##
## Df SS MS Rsq F Z Pr(>F)
## log(HaftW) 1 0.00574 0.0057382 0.01063 0.7738 0.14302 0.457
## Residuals 72 0.53390 0.0074153 0.98937
## Total 73 0.53964
##
## Call: procD.lm(f1 = coords ~ log(HaftW), iter = 999, RRPP = TRUE, data = CAHaftO.gdf,
## print.progress = FALSE)

Haft Shape ~ Haft-Length

fitOHL <- procD.lm(coords~ log(HaftL), data=CAHaftO.gdf, iter=999, RRPP=TRUE, print.progress = FALSE) # Regression of Haft shape on log normal Haft length
anova(fitOHL)# ANOVA Results for Haft length (Supplemental Table 4)

##
## Analysis of Variance, using Residual Randomization
## Permutation procedure: Randomization of null model residuals
## Number of permutations: 1000
## Estimation method: Ordinary Least Squares
## Sums of Squares and Cross-products: Type I
## Effect sizes (Z) based on F distributions
##
## Df SS MS Rsq F Z Pr(>F)
## log(HaftL) 1 0.04877 0.048770 0.09038 7.1535 2.6777 0.004 **
## Residuals 72 0.49087 0.006818 0.90962
## Total 73 0.53964
## ---
## Signif. codes: 0 '***' 0.001 '**' 0.01 '*' 0.05 '.' 0.1 ' ' 1
##
## Call: procD.lm(f1 = coords ~ log(HaftL), iter = 999, RRPP = TRUE, data = CAHaftO.gdf,
## print.progress = FALSE)

Figure 6 is created, a bivariate plot of Haft-width by Haft-length. Haft width sizes are colored and given different shapes. The bulk of those points are enclosed in rectangles. A LOWESS line and legend are created.

attach(CAHaftO.gdf)
par(mfcol = c(1,1)) # Creates single plot
plot(groupsO[,4:3],xaxt="none",yaxt="none", xlab="", ylab="") # Sets plot box and NON-CACHE points
lines(lowess(groupsO[,4:3]), col="black")
axis(1,seq(0,50,5)) #defines x-axis
axis(2,seq(0,45,5),las=2) #defines y-axis
minor.tick(nx = 10, ny = 5, tick.ratio=0.5) #minor ticks on axis, size and number
title(ylab="Haft Width", line=2.25) #y-axis label, size and position
title(xlab="Haft Length",line=2.25) #x-axis label, size and position
rect(11.5,16.5,27.5,24.5) # Small points rectangle
rect(11.5,29.5,40.5,24.5) # Medium points rectangle
rect(18.5,40.5,39.5,29.5) # Large points rectangle
rect(4.5,21.5,8.5,15.5) # Very small points rectangle
points(groupsO[which(Class=="S"),4:3],pch=21,cex=1,col="blue",bg="blue") # Color and shape of small size class
points(groupsO[which(Class=="M"),4:3],pch=22,cex=1,col="green",bg="green") # Color and shape of medium size class
points(groupsO[which(Class=="L"),4:3],pch=24,cex=1,col="red",bg="red") # Color and shape of large size class
points(groupsO[which(Class=="VS"),4:3],pch=3,cex=1,col="black") # Shape for very small points
legend(x="topleft", legend = c("Large","Medium","Small","Very Small"),col=c("red","green","blue","black"),pch=c(24,22,21,9), pt.bg=c("red","green","blue","black"))


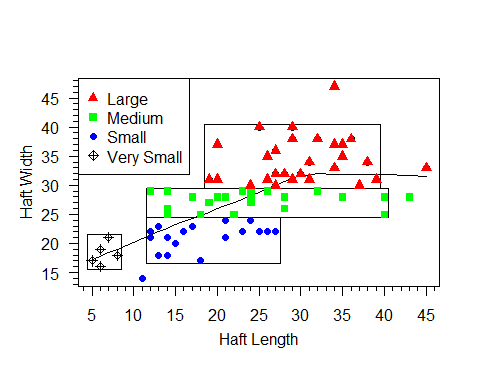


Figure 7 is created, a bivariate plot of Haft-width by blade-length. Haft width sizes are colored and given different shapes. The bulk of those points are enclosed in rectangles. A LOWESS line and legend are created.

plot(groupsO[,c(5,3)],xaxt="none",yaxt="none", xlab="", ylab="")
lines(lowess(groupsO[,c(5,3)]), col="black")
axis(1,seq(15,90,5))
axis(2,seq(0,50,5),las=2)
#mtext(side=3, line=0.2,"Scatterplot Haft Length and Blade Length", font=2, cex=1.5)
minor.tick(nx = 10, ny = 5, tick.ratio=0.5)
title(ylab="Haft Width", line=2.25)
title(xlab="Blade Length",line=2.25)
rect(20.5,15.5,40.5,24.5) # lower rectangle
rect(30.5,29.5,67.5,24.5) # middle rectangle
rect(41.5,40.5,90.5,29.5) # upper rectangle
points(groupsO[which(Class=="S"),c(5,3)],pch=21,cex=1,col="blue",bg="blue")
points(groupsO[which(Class=="M"),c(5,3)],pch=22,cex=1,col="green",bg="green")
points(groupsO[which(Class=="L"),c(5,3)],pch=24,cex=1,col="red",bg="red")
points(groupsO[which(Class=="VS"),c(5,3)],pch=3,cex=1,col="black")
legend(x="topleft", legend = c("Large","Medium","Small","Very Small"),col=c("red","green","blue","black"),pch=c(24,22,21,9), pt.bg=c("red","green","blue","black"))


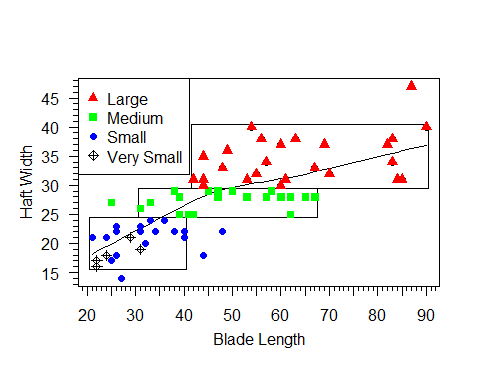


Figure 8 is created, a bivariate plot of Haft-length by blade-length. Haft width sizes are colored and given different shapes. The bulk of those points are enclosed in rectangles. A LOWESS line and legend are created.

plot(groupsO[,5:4],xaxt="none",yaxt="none", xlab="", ylab="")
lines(lowess(groupsO[,5:4]), col="black")
axis(1,seq(15,90,5))
axis(2,seq(0,45,5),las=2)
#mtext(side=3, line=0.2,"Scatterplot Haft Width and Blade Length", font=2, cex=1.5)
minor.tick(nx = 10, ny = 10, tick.ratio=0.5)
title(ylab="Haft Length", line=2.25)
title(xlab="Blade Length",line=2.25)
rect(23.5,10.25,44.5,19.5)
rect(30.5,19.5,61.5,28.5)
rect(52.5,28.5,87.5,40.5)
rect(21.5,8.5,31.5,4.5)
points(groupsO[which(Class=="S"),5:4],pch=21,cex=1,col="blue",bg="blue")
points(groupsO[which(Class=="M"),5:4],pch=22,cex=1,col="green",bg="green")
points(groupsO[which(Class=="L"),5:4],pch=24,cex=1,col="red",bg="red")
points(groupsO[which(Class=="VS"),5:4],pch=3,cex=1,col="black")
legend(x="topleft", legend = c("Large","Medium","Small","Very Small"),col=c("red","green","blue","black"),pch=c(24,22,21,9), pt.bg=c("red","green","blue","black"))


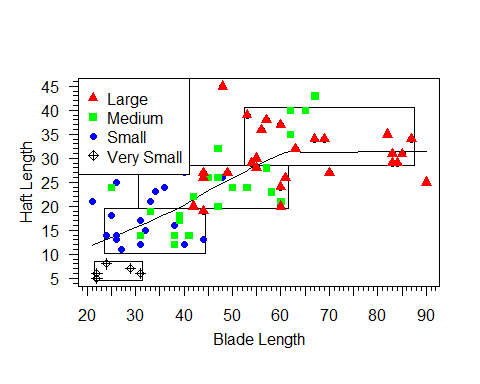
 END
