# Supplementary material for: Clovis point allometry, modularity, and integration: Exploring shape variation due to tool use with landmark-based geometric morphometrics
Source: PLoS One. 2023 Aug 16;18(8):e0289489. doi: 10.1371/journal.pone.0289489 (PMC10431674; doi:10.1371/journal.pone.0289489)
Supplement: S2 File — (ZIP) [file pone.0289489.s003.zip › S2_File_4.docx]

S3 File 4: Data Repositories.

Images used in the analyses were taken from high quality casts or original artifacts. Repositories for each artifact are listed in the Excel file CA Data1.xls. Contact information is provided below.

Smithsonian National Museum of Natural History

Department of Anthropology

4210 Silver Hill Road, Suitland, Maryland

Make appointments through <https://naturalhistory.si.edu/research/anthropology/collections-and-archives-access>

Denver Museum of Natural History and Science

2001 Colorado Blvd.

Denver Colorado 80205

Make arrangements through <https://www.dmns.org/science/anthropology/anthropology-collections/archaeology/#featuredStaff>

Arizona State Museum

1013 E. University Blvd.

Tucson Arizona

Suzanne L. Eckert, Ph.D.

Head of Collections

Arizona State Museum / University of Arizona

P.O. Box 210026

Tucson, AZ 85721-0026

520-626-0253

[sleckert@email.arizona.edu](mailto:sleckert@email.arizona.edu)

C. Vance Haynes Jr. Collection

Contact Vance Holliday [vthollid@email.arizona.edu](mailto:vthollid@email.arizona.edu)

School of Anthropology

University of Arizona

P.O. Box 210030

Tucson, AZ 85721-0030

Blackwater Draw Museum

bwdarchaeology@enmu.edu

Eastern New Mexico University

1500 S. Ave K, Lea Hall Room 163

Portales, NM 88130

Oklahoma University

Oklahoma Archaeological Survey

Lee Bement

lbement@ou.edu

111 East Chesapeake

Norman, Oklahoma 73019-5111

Gault Research Center

Gault School of Archaeological Research

Michael Collins

512-232-4912

P.O. Box 81563

Austin Texas 78708
