# Supplementary material for: Clovis point allometry, modularity, and integration: Exploring shape variation due to tool use with landmark-based geometric morphometrics
Source: PLoS One. 2023 Aug 16;18(8):e0289489. doi: 10.1371/journal.pone.0289489 (PMC10431674; doi:10.1371/journal.pone.0289489)
Supplement: S1 Table — Image Name, ID (tps file ID), Haft Width (HaftW), Haft Length (HaftL), Blade Length (BladeL), Maximum Length (MaxL), Cache/Non-cache, Class (Haft width size category), and Haft Width/Maximum Length ratio (SWML). (DOCX) [file pone.0289489.s004.docx]

**S1 Table.** **Details about each of the points used in the analyses. Image Name, ID (tps file ID), Haft Width (HaftW), Haft Length (HaftL), Blade Length (BladeL), Maximum Length (MaxL), Cache/Non-cache, Class (Haft width size category), and Haft Width/Maximum Length ratio (SWML).**

| **Image** | **ID** | **HaftW** | **HaftL** | **BladeL** | **MaxL** | **Cache** | **Class** |
| --- | --- | --- | --- | --- | --- | --- | --- |
| AZESC1 | 0 | 38 | 32 | 63 | 95 | Other | L |
| AZLEH1 | 1 | 30 | 24 | 60 | 84 | Other | L |
| AZLEH2 | 3 | 30 | 37 | 60 | 97 | Other | L |
| AZLEH3 | 4 | 31 | 19 | 44 | 63 | Other | L |
| AZLEH4 | 5 | 25 | 14 | 41 | 55 | Other | M |
| AZLEH5 | 6 | 29 | 14 | 38 | 52 | Other | M |
| AZLEH6 | 7 | 19 | 6 | 31 | 37 | Other | VS |
| AZLEH7 | 8 | 18 | 8 | 24 | 32 | Other | VS |
| AZLEH8 | 9 | 20 | 15 | 32 | 47 | Other | S |
| AZLEH9 | 10 | 28 | 21 | 60 | 81 | Other | M |
| AZLEH10 | 2 | 29 | 24 | 50 | 74 | Other | M |
| AZLEK1 | 11 | 33 | 45 | 48 | 93 | Other | L |
| AZMSP1 | 12 | 35 | 35 | 37 | 72 | Other | L |
| AZMSP2 | 13 | 21 | 21 | 21 | 42 | Other | S |
| AZMSP3 | 14 | 27 | 24 | 25 | 49 | Other | M |
| AZNAC1 | 15 | 32 | 28 | 55 | 83 | Other | L |
| AZNAC2 | 16 | 29 | 26 | 47 | 73 | Other | M |
| AZNAC3 | 17 | 35 | 26 | 44 | 70 | Other | L |
| AZNAC4 | 18 | 26 | 28 | 31 | 59 | Other | M |
| AZNAC5 | 19 | 32 | 27 | 70 | 97 | Other | L |
| AZNAC6 | 20 | 28 | 35 | 62 | 97 | Other | M |
| AZSHK1 | 21 | 23 | 17 | 31 | 48 | Other | S |
| CODEN1 | 22 | 34 | 31 | 83 | 114 | Other | L |
| CODEN2 | 23 | 38 | 36 | 56 | 92 | Other | L |
| CODRK1 | 24 | 39 | 27 | 90 | 117 | Cache | C |
| CODRK2 | 28 | 36 | 40 | 97 | 137 | Cache | C |
| CODRK3 | 29 | 35 | 24 | 68 | 92 | Cache | C |
| CODRK4 | 30 | 43 | 31 | 130 | 161 | Cache | C |
| CODRK5 | 31 | 37 | 31 | 60 | 91 | Cache | C |
| CODRK6 | 32 | 39 | 27 | 121 | 148 | Cache | C |
| CODRK7 | 33 | 36 | 35 | 76 | 111 | Cache | C |
| CODRK8 | 34 | 39 | 25 | 81 | 106 | Cache | C |
| CODRK9 | 35 | 34 | 35 | 78 | 113 | Cache | C |
| CODRK10 | 25 | 42 | 26 | 91 | 117 | Cache | C |
| CODRK11 | 26 | 36 | 25 | 100 | 125 | Cache | C |
| CODRK12 | 27 | 38 | 27 | 104 | 131 | Cache | C |
| COFOX1 | 36 | 40 | 29 | 54 | 83 | Other | L |
| COGLY1 | 37 | 37 | 35 | 82 | 117 | Other | L |
| COKGP1 | 38 | 47 | 34 | 87 | 121 | Other | L |
| IARMK1 | 39 | 35 | 34 | 73 | 107 | Cache | C |
| IARMK2 | 40 | 29 | 34 | 51 | 85 | Cache | C |
| IARMK3 | 41 | 36 | 38 | 65 | 103 | Cache | C |
| I.D.SIM1 | 42 | 36 | 18 | 76 | 94 | Cache | C |
| I.D.SIM2 | 43 | 40 | 37 | 122 | 159 | Cache | C |
| I.D.SIM3 | 44 | 40 | 43 | 142 | 185 | Cache | C |
| I.D.SIM4 | 45 | 39 | 22 | 164 | 186 | Cache | C |
| MOKIM1 | 46 | 25 | 40 | 62 | 102 | Other | M |
| MOKIM2 | 47 | 22 | 25 | 26 | 51 | Other | S |
| MTANZ1 | 48 | 37 | 42 | 123 | 165 | Cache | C |
| MTANZ2 | 49 | 39 | 37 | 69 | 106 | Cache | C |
| MTANZ3 | 50 | 32 | 38 | 47 | 85 | Cache | C |
| MTANZ4 | 51 | 32 | 43 | 51 | 94 | Cache | C |
| MTANZ5 | 52 | 29 | 27 | 52 | 79 | Cache | C |
| NMBWD1 | 53 | 18 | 13 | 44 | 57 | Other | S |
| NMBWD2 | 62 | 18 | 14 | 26 | 40 | Other | S |
| NMBWD3 | 63 | 21 | 7 | 29 | 36 | Other | VS |
| NMBWD4 | 64 | 17 | 5 | 22 | 27 | Other | VS |
| NMBWD5 | 65 | 14 | 11 | 27 | 38 | Other | S |
| NMBWD6 | 66 | 16 | 6 | 22 | 28 | Other | VS |
| NMBWD7 | 67 | 38 | 29 | 83 | 112 | Other | L |
| NMBWD8 | 68 | 28 | 28 | 57 | 85 | Other | M |
| NMBWD9 | 69 | 22 | 12 | 31 | 43 | Other | S |
| NMBWD10 | 54 | 28 | 43 | 67 | 110 | Other | M |
| NMBWD11 | 55 | 28 | 40 | 65 | 105 | Other | M |
| NMBWD12 | 56 | 36 | 27 | 49 | 76 | Other | L |
| NMBWD13 | 57 | 37 | 34 | 69 | 103 | Other | L |
| NMBWD14 | 58 | 23 | 13 | 26 | 39 | Other | S |
| NMBWD15 | 59 | 24 | 21 | 33 | 54 | Other | S |
| NMBWD16 | 60 | 21 | 12 | 40 | 52 | Other | S |
| NMBWD17 | 61 | 29 | 12 | 38 | 50 | Other | M |
| NMBWD18 | 70 | 26 | 14 | 31 | 45 | Other | M |
| OKDOB1 | 71 | 22 | 27 | 40 | 67 | Other | S |
| OKDOB2 | 72 | 29 | 32 | 47 | 79 | Other | M |
| OKJBF1 | 73 | 27 | 19 | 33 | 52 | Other | M |
| OKJBF2 | 74 | 17 | 18 | 25 | 43 | Other | S |
| ORDTZ1 | 75 | 32 | 30 | 55 | 85 | Other | L |
| SDLGF1 | 76 | 22 | 16 | 38 | 54 | Other | S |
| SDLGF2 | 77 | 21 | 14 | 24 | 38 | Other | S |
| TXGLT1 | 78 | 24 | 24 | 36 | 60 | Other | S |
| TXGLT2 | 80 | 22 | 23 | 34 | 57 | Other | S |
| TXGLT3 | 81 | 25 | 18 | 39 | 57 | Other | M |
| TXGLT4 | 82 | 31 | 39 | 53 | 92 | Other | L |
| TXGLT5 | 83 | 28 | 24 | 53 | 77 | Other | M |
| TXGLT6 | 84 | 31 | 26 | 61 | 87 | Other | L |
| TXGLT7 | 85 | 25 | 22 | 42 | 64 | Other | M |
| TXGLT8 | 86 | 30 | 27 | 44 | 71 | Other | L |
| TXGLT9 | 87 | 22 | 26 | 48 | 74 | Other | S |
| TXGLT10 | 79 | 33 | 38 | 63 | 101 | Other | L |
| TXMIA1 | 88 | 31 | 31 | 85 | 116 | Other | L |
| TXMIA2 | 89 | 31 | 29 | 84 | 113 | Other | L |
| WAEWS1 | 90 | 62 | 43 | 188 | 231 | Cache | C |
| WAEWS2 | 91 | 64 | 48 | 184 | 232 | Cache | C |
| WYCBY1 | 92 | 28 | 17 | 39 | 56 | Other | M |
| WYCBY2 | 93 | 34 | 38 | 57 | 95 | Other | L |
| WYCBY3 | 94 | 31 | 20 | 42 | 62 | Other | L |
| WYCKM1 | 95 | 29 | 23 | 58 | 81 | Other | M |
| WYCSP1 | 96 | 29 | 26 | 45 | 71 | Other | M |
| WYHEL1 | 97 | 40 | 25 | 90 | 115 | Other | L |
| WYHEL2 | 98 | 37 | 20 | 60 | 80 | Other | L |
| WYSHE1 | 99 | 28 | 20 | 47 | 67 | Other | M |
