# Supplementary material for: Clovis point allometry, modularity, and integration: Exploring shape variation due to tool use with landmark-based geometric morphometrics
Source: PLoS One. 2023 Aug 16;18(8):e0289489. doi: 10.1371/journal.pone.0289489 (PMC10431674; doi:10.1371/journal.pone.0289489)
Supplement: S2 Table — (ZIP) [file pone.0289489.s005.zip › S2_Table_1.docx]

**S2 Table 1: ANOVA results for regressions of the entire point, stem, and blade shapes on the natural logarithms of their respective centroid sizes ln(CSE), ln(CSS), ln(CSB)**. All ANOVAs nonparametric using RRPP randomizing null model residuals with 10,000 permutations. Ordinary Least Squares estimation method; Type I linear model. Effect sizes (Z) based on F distributions. α=.05. Results rounded to 3 decimal places.

|  | DF | SS | MS | r^2^ | F | Z | Pr(>F) |
| --- | --- | --- | --- | --- | --- | --- | --- |
| ln(CSE) | 1 | 0.475 | 0.475 | 0.360 | 55.217 | 6.304 | 9.999e-05 |
| Residuals | 98 | 0.843 | 0.009 | 0.640 |  |  |  |
| Total | 99 | 1.317 |  |  |  |  |  |
|  |  |  |  |  |  |  |  |
| ln(CSS) | 1 | 0.165 | 0.165 | 0.216 | 26.945 | 5.0369 | 9.999e-05 |
| Residuals | 98 | 0.601 | 0.006 | 0.784 |  |  |  |
| Total | 99 | 0.766 |  |  |  |  |  |
|  |  |  |  |  |  |  |  |
| ln(CSB) | 1 | 0.229 | 0.229 | 0.416 | 69.674 | 6.633 | 9.999e-05 |
| Residuals | 98 | 0.322 | 0.003 | 0.584 |  |  |  |
| Total | 99 | 0.551 |  |  |  |  |  |

Df=degrees of freedom, SS=sum of squares, MS=mean square, r^2^= coefficient of determination, F=F score, Z=Z score, Pr(>F) = p-value.
