# Supplementary material for: Clovis point allometry, modularity, and integration: Exploring shape variation due to tool use with landmark-based geometric morphometrics
Source: PLoS One. 2023 Aug 16;18(8):e0289489. doi: 10.1371/journal.pone.0289489 (PMC10431674; doi:10.1371/journal.pone.0289489)
Supplement: S2 Table — (ZIP) [file pone.0289489.s005.zip › S2_Table_2.docx]

**S2 Table 2:** **ANOVA results for regressions of the entire point, blade, and stem shapes between cache and non-cache points**. All ANOVAs nonparametric using RRPP randomizing null model residuals with 10,000 permutations. Ordinary Least Squares estimation method; Type I linear model. Effect sizes (Z) based on F distributions. α=.05. Results rounded to 3 decimal places.

|  | DF | SS | MS | r^2^ | F | Z | Pr(>F) |
| --- | --- | --- | --- | --- | --- | --- | --- |
| Entire | 1 | 0.194 | 0.194 | 0.147 | 16.872 | 4.129 | 0.001 |
| Residuals | 98 | 1.124 | 0.011 | 0.853 |  |  |  |
| Total | 99 | 1.317 |  |  |  |  |  |
|  |  |  |  |  |  |  |  |
| Blade | 1 | 0.104 | 0.104 | 0.189 | 22.779 | 4.366 | 0.001 |
| Residuals | 98 | 0.447 | 0.005 | 0.811 |  |  |  |
| Total | 99 | 0.551 |  |  |  |  |  |
|  |  |  |  |  |  |  |  |
| Stem | 1 | 0.090 | 0.090 | 0.117 | 12.966 | 3.765 | 0.001 |
| Residuals | 98 | 0.677 | 0.007 | 0.883 |  |  |  |
| Total | 99 | 0.766 |  |  |  |  |  |

Df=degrees of freedom, SS=sum of squares, MS=mean square, r^2^= coefficient of determination, F=F score, Z=Z score, Pr(>F) = p-value.
