# Supplementary material for: Clovis point allometry, modularity, and integration: Exploring shape variation due to tool use with landmark-based geometric morphometrics
Source: PLoS One. 2023 Aug 16;18(8):e0289489. doi: 10.1371/journal.pone.0289489 (PMC10431674; doi:10.1371/journal.pone.0289489)
Supplement: S2 Table — (ZIP) [file pone.0289489.s005.zip › S2_Table_3.docx]

**S2 Table 3. Analysis of Variance, using Residual Randomization reports results supporting Figure 5 in text.** Permutation procedure: Randomization of null model residuals; number of permutations: 1000; Estimation method: Ordinary Least Squares; Effect sizes (Z) based on F distributions. The tests compare the common (the null hypothesis) and unique allometries for cache and non-cache points. Allometries are tested as shape v. ln centroid size of the entire (E-S), blade (B-S), and stem (S-S) shapes. α = .05. Results rounded to 3 decimal places.

|  | ResDF | Df | RSS | SS | MS | r^2^ | F | Z | Pr(>F) |
| --- | --- | --- | --- | --- | --- | --- | --- | --- | --- |
| E-S ~ lnCS + Cache (Null) | 97 | 1 | 0.810 |  |  | 0.000000 |  |  |  |
| E-S ~ lnCS * Cache | 96 | 1 | 0.735 | 0.075 | 0.075 | 0.057 | 9.843 | 3.225 | 0.002 |
| Total | 99 |  | 1.319 |  |  |  |  |  |  |
