# Supplementary material for: Clovis point allometry, modularity, and integration: Exploring shape variation due to tool use with landmark-based geometric morphometrics
Source: PLoS One. 2023 Aug 16;18(8):e0289489. doi: 10.1371/journal.pone.0289489 (PMC10431674; doi:10.1371/journal.pone.0289489)
Supplement: S2 Table — (ZIP) [file pone.0289489.s005.zip › S2_Table_4.docx]

**S2 Table 4. ANOVA results for regressions of haft shape for non-cache Clovis points on haft size-class, and natural logarithms of haft length, width**. All ANOVAs nonparametric using RRPP randomizing null model residuals with 10,000 permutations. Ordinary Least Squares estimation method; Type I linear model. Effect sizes (Z) based on F distributions. α=.05. Results rounded to 3 decimal places.

|  | DF | SS | MS | r^2^ | F | Z | Pr(>F) |
| --- | --- | --- | --- | --- | --- | --- | --- |
| Size Class | 3 | 0.025 | 0.008 | 0.046 | 1.121 | 0.397 | 0.354 |
| Residuals | 70 | 0.515 | 0.007 | 0.954 |  |  |  |
| Total | 73 | 0.540 |  |  |  |  |  |
|  |  |  |  |  |  |  |  |
| ln Haft-length | 1 | 0.049 | 0.049 | 0.090 | 7.154 | 2.678 | 0.004 |
| Residuals | 72 | 0.491 | 0.007 | 0.910 |  |  |  |
| Total | 73 | 0.540 |  |  |  |  |  |
|  |  |  |  |  |  |  |  |
| ln Haft-width | 1 | 0.006 | 0.006 | 0.011 | 0.773 | 0.143 | 0.457 |
| Residuals | 72 | 0.534 | 0.007 | 0.989 |  |  |  |
| Total | 73 | 0.540 |  |  |  |  |  |

DF=degrees of freedom, SS=sum of squares, MS=mean square, r^2^= coefficient of determination, F=F score, Z=Z score, Pr(>F) = p-value.
